# Solid-state cooling by elastocaloric polymer with uniform chain-lengths

**DOI:** 10.1038/s41467-021-27746-y

**Published:** 2022-01-10

**Authors:** Shixian Zhang, Quanling Yang, Chenjian Li, Yuheng Fu, Huaqing Zhang, Zhiwei Ye, Xingnan Zhou, Qi Li, Tao Wang, Shan Wang, Wenqing Zhang, Chuanxi Xiong, Qing Wang

**Affiliations:** 1 State Key Laboratory of Advanced Technology for Materials Synthesis and Processing, and School of Materials Science and Engineering, Wuhan University of Technology, 430070 Wuhan, China; 2 Department of Electrical Engineering, Tsinghua University, 100084 Beijing, China; 3 Wuhan Optics Valley United Property Rights Exchange, 430015 Wuhan, China; 4 Department of Materials Science and Engineering, The Pennsylvania State University, University Park, PA USA

**Keywords:** Polymers, Organic molecules in materials science, Polymers

## Abstract

Although the elastocaloric effect was found in natural rubber as early as 160 years ago, commercial elastocaloric refrigeration based on polymer elastomers has stagnated owing to their deficient elastocaloric effects and large extension ratios. Herein, we demonstrate that polymer elastomers with uniform molecular chain-lengths exhibit enormous elastocaloric effects through reversible conformational changes. An adiabatic temperature change of −15.3 K and an isothermal entropy change of 145 J kg^−1^ K^−1^, obtained from poly(styrene-b-ethylene-co-butylene-b-styrene) near room temperature, exceed those of previously reported elastocaloric polymers. A rotary-motion cooling device is tailored to high-strains characteristics of rubbers, which effectively discharges the cooling energy of polymer elastomers. Our work provides a strategy for the enhancement of elastocaloric effects and could promote the commercialization of solid-state cooling devices based on polymer elastomers.

## Abstract (web summary)

The elastocaloric effect was originally found in natural rubber, but the development of polymer based elastocaloric cooling still remains underdeveloped. Herein, the authors demonstrate that polymer elastomers with uniform molecular chain-lengths exhibit enormous elastocaloric effects through reversible conformational changes.

## Introduction

Current commercial and residential cooling devices, such as air conditioners, refrigerators, etc., are mostly based on vapor-compression technology^1^. The releases of gas refrigerants used in this mature technology have generated and exacerbated the global climate change, e.g., global warming^2–4^. Solid-state cooling technology based on caloric effects (CEs) has the potential to revolutionize vapor-compression refrigeration technology owing to its high energy efficiency and zero-greenhouse gas emissions^1–3^. CEs, as the core principle of solid-state cooling technology, are typically characterized by an adiabatic temperature change (Δ*T*_adi_) and an isothermal entropy change (Δ*S*_iso_) when the working material undergoes a reversible first-order phase transition on the application or removal of an external field^5–8^. CEs were first investigated by Joule with natural rubber (NR) as the research object^9^. At present, CEs can be classified into magnetocaloric, electrocaloric, elastocaloric, barocaloric and twistocaloric effects depending on the nature and mode of action of the external field^10–15^. In particular, due to the enormous elastocaloric effects (E-CEs) found in the martensitic transition of shape-memory alloys (SMAs) around room temperature^16–20^, solid-state cooling technology based on E-CEs is considered to be the best promising alternative to conventional refrigeration devices^21–24^. However, the corresponding tensile stress (*σ*) values of SMAs can reach several hundreds of megapascal, which remains a challenging issue for practical application (Supplementary Table 1).

The *σ* of a polymer working medium is one or two orders of magnitudes smaller than those applied to SMAs, but the commercialization of elastocaloric refrigeration based on polymer-tension technology is obstructed by their low E-CEs. Enhanced E-CEs via strain-induced crystallization (SIC) in NR at room temperature were reported recently^25–27^. Polyvinylidene difluoride based on the *α*-*β* phase transition also shows promising E-CEs^28,29^. Nevertheless, the regular arrangement of polymer molecular chains into a lattice is constrained due to the relatively high molecular weight, resulting in restricted crystalline rates with limited latent heat contributions. In contrast, E-CEs caused by reversible conformational changes of polymer molecular chains are independent of the amorphous-crystalline transition. When a single polymer molecular chain transitions from curled to straight, large conformational adjustments are required for this process. The conformational changes of the integrated network chains in a polymer working medium from curled to straight would be astronomical, which would result in enormous E-CEs. However, the conformational changes during the uniaxial tensile processes of elastomers are constrained by the uniformity of the molecular chain-length. Therefore, we speculate that an effective path to enhancing the E-CEs in polymer elastomers is to improve the uniformity of the polymer molecular chain-length.

In this study, a series of commercial triblock poly(styrene-b-ethylene-co-butylene-b-styrene) (SEBS) thermoplastic elastomers (TPEs), with polystyrene (PS) blocks (≈30 wt%) surrounding a central block of poly(ethylene-co-butylene) (PE/PB) segments (≈70 wt%), were selected to demonstrate the dependence of the E-CEs on the chain-length uniformity in SEBS (“Methods”). Furthermore, a rotary-motion cooling device was tailored to high-strains characteristics of rubbers, which effectively discharged the cooling energy of polymer elastomers.

## Results

### E-CEs in SEBS films

**Fig. 1:**
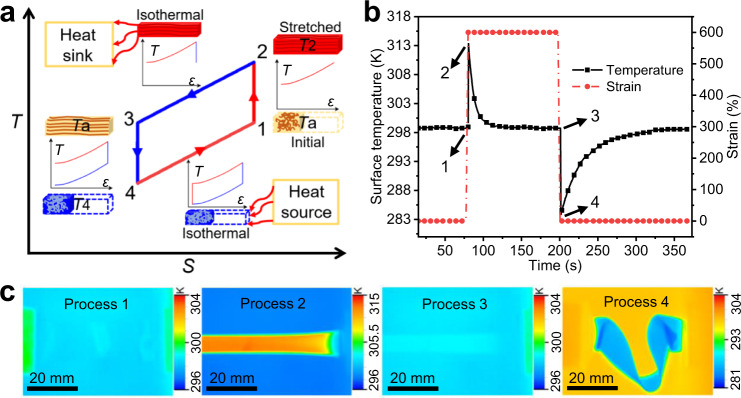
E-CE cycle and temperature change of TPEs-1. **a** Schematic *T*-*S* diagram of the E-CE cooling cycle. The insets show the variations of the sample temperature as a function of strain. During stage 1–2, the sample is stretched adiabatically by a uniaxial tension, increasing the surface temperature (*T*_a_ → *T*_2_). During stage 2–3, the strain remains constant, and the thermal entropy decreases isothermally until *T* = *T*_a_. During stage 3–4, the specimen retracts adiabatically with the unloading of the tension, further decreasing the temperature of the specimen (*T*_a_ → *T*_4_). During stage 4–1, the thermal entropy of the material increases until *T* = *T*_a_. **b** Typical strain evolution (analogized from the displacement of the slide table) and the resulting temperature variation of TPEs-1 during a single E-CE cycle. **c** Infrared thermal images of different processes recorded using an infrared thermal imager.

The molecular properties and sample labeling of these SEBS samples are summarized in Supplementary Table 2 and Supplementary Fig. 1. The Δ*T*_adi_ associated with the E-CEs in the TPEs was induced under a uniaxial-strain-controlled system in an open indoor environment (Methods, Supplementary Fig. 2). Thus, the simplification of the ideal elastocaloric cooling cycle could be analogically derived from the reverse Brayton cycle as shown in Fig. 1a^30^. To quantify the E-CEs, Δ*T*_adi_ can be directly measured by an infrared thermometer when TPEs undergo a strain rate (15 s^−1^) higher than the adiabatic strain rate (Supplementary Discussion 1, Supplementary Fig. 4). Infrared measurements provide the resulting temperature variations of TPEs with the narrowest molecular weight distribution (TPEs-1) during a single elastocaloric cooling cycle (Fig. 1b, c). During the adiabatic stretching process, the surface temperature of the TPEs-1 was found to increase significantly from the ambient temperature *T*_a_ = 299.0 K to the temperature *T*_2_ = 313.5 K when the strain abruptly increased from 0% to 600%. During the exothermic process, the surface temperature decreased exponentially to room temperature (Supplementary Fig. 5). When the TPEs-1 sample returned to the initial length, the surface temperature dropped from *T*_a_ = 299.0 K to *T*_4_ = 283.7 K, which corresponding to a Δ*T*_adi_ = −15.3 K. Finally, TPEs-1 returned to room temperature through an isothermal endothermic process.

### Origin of E-CE entropy change and its influencing factors

**Fig. 2:**
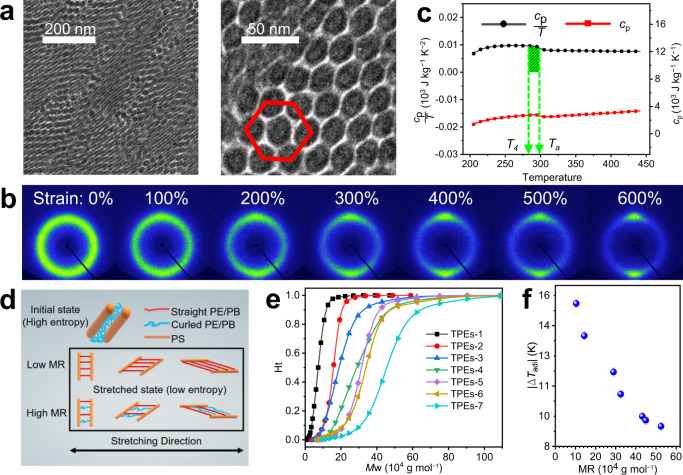
Origin of E-CE entropy change and its influencing factors. **a** TEM images of TPEs-1 at a strain of 0%. PS domains (black regions) were stained dark by RuO_4_, and the light regions correspond to PE/PB domains. **b** 2D WAXD diffractograms of TPEs-1 at different strain levels. **c** Heat capacity *c*_*p*_ and *c*_*p*_/*T* versus temperature during the heating process of TPEs-1. The green shadow represents the integral region of *c*_*p*_/*T* from *T*_4_ to *T*_a_. **d** Schematic diagram of PE/PB conformational evolution in the stretching process of TPEs with different MRs. The red fibers, blue fibers and orange cylinders represent straight PE/PB segments, curled PE/PB segments and PS segments, respectively. **e** GPC curves of TPEs with different MRs. **f** |Δ*T*_adi_| versus MR of TPEs within the ultimate strains (former one before fracture strain) during the cooling process.

In the initial state, PS domains with hexagonal packed cylindrical (HPC) microstructures were randomly embedded in the amorphous PE/PB matrix (Fig. 2a). PS hard segments, as physical crosslinking points, and PE/PB soft segments, as the elastic matrix, were linked by chemical bonds to transfer applied tension. As shown in Fig. 2b, a wide and bright dispersion ring of the unstretched TPEs-1 appeared near *q* = 1.33 Å^−1^. The relative intensity of the dispersion ring along the stretching direction diminished as the strain increased, while that perpendicular to stretching direction was continuously concentrated and increased without any indication of crystallization. The two-dimensional wide-angle X-ray diffraction (2D WAXD) pattern of TPEs-1 returned to the wide dispersion ring when the strain returned to the initial state (Supplementary Fig. 6). This indicated that during the stretching–recovery cycle, the amorphous PE/PB matrix only underwent reversible transitions from curled chain conformations to oriented chain conformations along the stretching direction without any phase transition such as SIC (details in Supplementary Discussion 2, Supplementary Figs. 7–10). The Δ*S*_iso_ of TPEs-1 at *T*_a_ = 298 K was estimated to be 145 J kg^−1^ K^−1^ by integrating $\frac{{c}_{{{{{{\rm{p}}}}}}}}{T}$ from *T*_4_ to *T*_a_ (Fig. 2c, Supplementary Eq. (12)), where *c*_*p*_ is the specific heat capacity of TPEs-1. This superior Δ*S*_iso_ value stemmed from the excellent uniformity of the molecular chain-length in TPEs-1.

Upon tensile stress, shorter molecular chains in the TPEs network would be straightened first, which would inhibit the further stretching of longer molecular chains (Fig. 2d) and consequently obstruct some conformational transformations. These obstructed conformational transitions resulted in lower apparent values of the E-CE. The obstructed conformational transitions were related to the uniformity of the molecular chain-lengths. The uniformity of the molecular chain-length can be characterized by the molecular weight distribution range (MR), which is defined as the difference value between the maximum and the minimum molecular weights of the complete molecular chains in a sample. Gel permeation chromatography (GPC) analysis indicated that the seven samples possessed different MRs (Supplementary Table 2, Fig. 2e). TPEs-1 possessed the smallest MR, suggesting that its molecular chain-length was the most uniform. As illustrated in Fig. 2f and Supplementary Figs. 18, 19, the |Δ*T*_adi_| of the TPEs decreased from 15.3 to 9.0 K with the increase in MR, indicating that the higher the MR was, the more obstructed the conformational transitions in the TPEs became. As shown in Supplementary Fig. 11b, even for TPEs-1, with the most uniform chain-length, the theoretical E-CE (|Δ*T*_adi_| = 23.6 K at a strain of 600%) was also much higher than the experimental E-CE under a large strain, highlighting the importance of reducing the obstructed conformational transitions by improving the uniformity of the molecular chain-length (details in Supplementary Discussion 3).

### Material properties

**Fig. 3:**
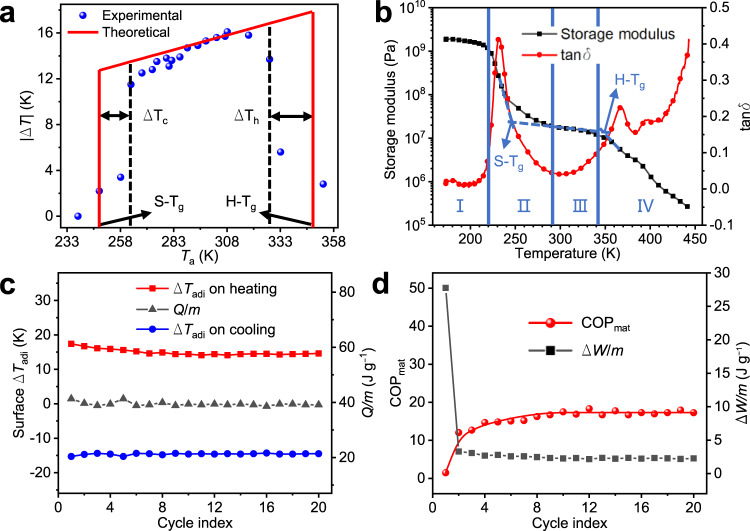
Ambient temperature range and COP_mat_ of TPEs-1. **a** Experimental and theoretical |Δ*T*| of TPEs-1 as a function of the ambient temperature under the ultimate strains during the cooling process. The theoretical change of |Δ*T*_adi_| is proportional to *T*_a_ when the elongation ratio (*λ*) and *c*_*p*_ are constant, and the slope is estimated as the ratio of |Δ*T*_adi_| = 15.3 K to *T*_a_ = 298.15 K according to Supplementary Eq. (4) in Supplementary Discussion 3. **b** *E’* and tan δ versus temperature curves of TPEs-1. Region I is the glass state region of TPEs-1. Region II is the glass transition region of the soft segments. Region III is the rubber elastic plateau region. Region IV is the viscous flow region. **c** Surface Δ*T*_adi_ evolution of TPEs-1 during 20 times of elastocaloric cycles with 600% strain. **d** COP_mat_ and input work of unit mass in TPEs-1 during elastocaloric cycles.

TPEs can produce enormous E-CEs over a wide *T*_a_ range due to their broad rubber elastic plateau region (Fig. 3a, b). For a temperature range of 238–353 K, |Δ*T*_adi_| exhibited a strong dependence on *T*_a_. In the glass transition region of the PE/PB segment (*T*_a_ = 263–293 K, region II in Fig. 3b), the storage modulus (*E’*) of TPEs-1 increased with the decrease in temperature, which indicated that the PE/PB soft segments began to stiffen and the conformational transition became more difficult. Thus, the experimental value of |Δ*T*_adi_| in this region was lower than the theoretical value. This can also be evidenced by comparing Young’s modulus (*E*) values at different stretching temperatures (Supplementary Discussion 4, Supplementary Fig. 20). When *T*_a_ was below 263 K, the sample temperature was further reduced (Δ*T*_c_ in Fig. 3a) owing to the recovery cooling, which caused the specimen temperature to reach a value closer to the glass transition temperature of the soft segments (S-T_g_ ≈ 247 K) and resulted in the freezing of elastic soft segments. It is reasonable that the recovery strain rate of the freezing segments dropped below the adiabatic strain rate, so that the effect of heat transfer caused by convection and radiation between TPEs-1 and the ambient environment was enhanced, which led to a significant drop in |Δ*T*_adi_|. This drop in |Δ*T*_adi_| was also observed when *T*_a_ increased above 328 K, which was related to the destruction of the crosslinking points at the glass transition temperature of hard segments (H-*T*_g_). When *T*_a_ increased to 328 K, the sample temperature further increased (Δ*T*_h_ in Fig. 3a) due to the tensile heating, which caused the specimen temperature to enter the region IV in Fig. 3b. In this region, the destruction of the PS crosslinking points led to the slip of the whole molecular chains accompanied by the irreversible deformation under tensile stress. Nonetheless, in the wide ambient temperature range of 263–328 K, TPEs-1 could yield a |Δ*T*_adi_| of about 11.5–16.1 K.

The material coefficient of performance (COP_mat_) for TPEs near room temperature is described by the ratio of the cooling energy per unit mass (*Q/m*) to the input work per unit mass (Δ*W/m*) (details in Supplementary Discussion 5, Supplementary Eq. (13)). As illustrated in Supplementary Discussion 6, all TPEs generally exhibit stable cyclic behavior after their first 20 cycles. The cyclic behaviors of the first 20 times of E-CE cycles for TPEs-1 are shown in Fig. 3c. It is encouraging that the Δ*T*_adi_ and the *Q/m* of TPEs-1 remained constant during 20 cycles, explaining its outstanding and steady cycling performance. Nevertheless, TPEs-1 showed an extremely low COP_mat_ value in the 1st cycle (Fig. 3d), which was mainly due to the large Δ*W/m*. As is well known, there are high internal stresses in polymer elastomer products, which are generated during the molding process, and it takes considerable work to eliminate these internal stresses. Gratifyingly, TPEs-1 exhibited an exceedingly stable COP_mat_ value after the first cycle, suggesting that a single stretch could basically eliminate the internal stress of TPEs-1. The average COP_mat_ of TPEs-1 during the stable cycles (2nd to 20th) was estimated to be 16.2 by Supplementary Eq. (13) (details in Supplementary Discussion 5, Supplementary Fig. 21).

### Rotary-motion cooling device

**Fig. 4:**
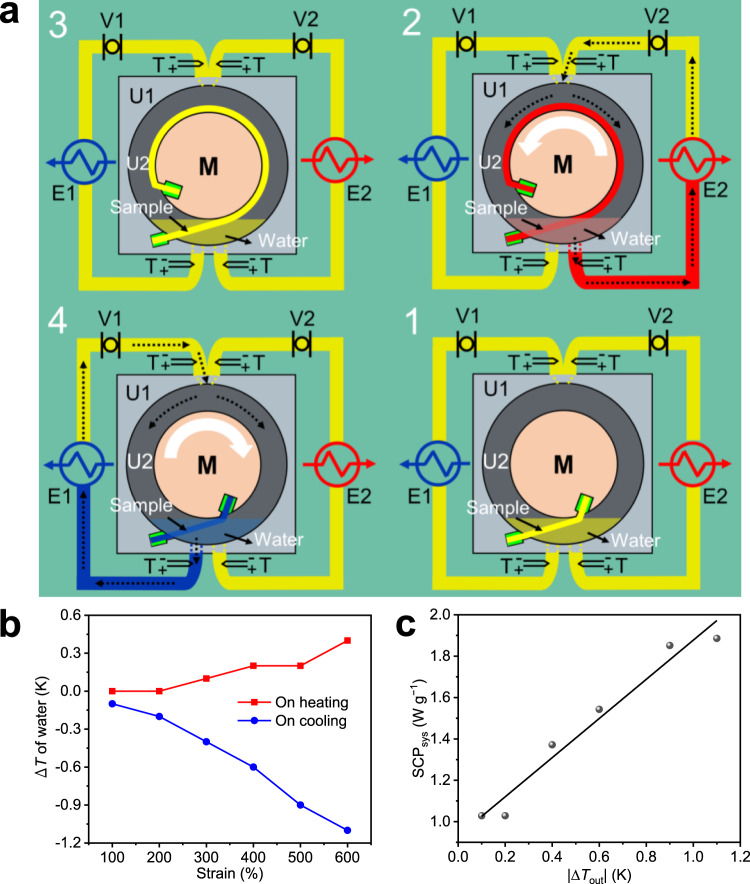
Principle and performance of rotary motion cooling device. **a** Schematic diagram of the cooling device. Stage 1: one end of sample is fixed on the body case (U1), and the other is fixed to the revolving disc (M). Stage 2: M rotates counterclockwise causing the elongation of sample. Water flows toward the hot heat exchanger (E2) to release heat to the environment. Stage 3: The sample maintains elongation until the sample and water temperature recover to *T*_a_. Stage 4: M rotates clockwise causing the contraction of sample. Water flows toward the cold heat exchanger (E1) to absorb heat from the environment. The symbols V1 and V2 represent precision pumps. The symbols T represent thermocouples. U2 is a cavity that allows water flow and sample dilation. The mass of water in U2 is constant. The black dotted arrow points to the flow direction of the water. The white arrow points to the rotation direction of M. **b** Temperature changes of outlet water versus strain after a single cycle. The strain of sample was adjusted by controlling the rotation angle of M. **c** The SCP_sys_ vs. Δ*T*_out_ of the rotary motion elasto-based cooling device.

The E-CE results (Δ*T*_adi_ ≈ −15.3 K, Δ*S*_iso_ ≈ 145 J kg^−1^ K^−1^) of TPEs-1 at room temperature exceeded those for all previously reported elastocaloric polymers (Supplementary Fig. 37), and thus, it is a feasible material for cooling devices. However, large deformation spaces are inherent for polymer elastomers (Supplementary Table 1). A rotary-motion cooling device based on the E-CE of TPEs-1 allowed a single cooling cycle to be designed for heat separation and output under high-strain with continuously flowing water (Fig. 4a, details in Supplementary Discussion 7 and Supplementary Fig. 38). The water current with room temperature flowed into the cavity from the top inlet over TPEs-1, and removing 600% of the strain cooled the outlet water by −1.1 K (Fig. 4b). According to Supplementary Eq. (14), The COP_sys_ for the cooling process of the full system was estimated as 9.3. On the other hand, as the strain amplitude increased from 100% to 600% during the cooling processes, the temperature change of the outlet water (|Δ*T*_out_|) increased from 0.1 K to 1.1 K (Fig. 4b). In this process (no temperature span), the cooling power (SCP_sys_) of the system was determined by the temperature change of the cold water. As shown in Fig. 4c, the corresponding SCP_sys_ of the rotary motion cooling device increased almost linearly with the increase of |Δ*T*_out_| (SCP_sys_ values were calculated by Supplementary Eq. (15)). And the maximum value of SCP_sys_ was about 1.9 W g^−1^. The comparison among the obtained SCP_sys_ and that reported in the literature is summarized in Supplementary Table 4. Furthermore, the cold heat exchanger (E1) was replaced by a heat storage tank (details in Supplementary Discussion 7). After 10th cooling cycles with a constant strain amplitude of 600%, the thermal loss was stable and the system achieved a maximum temperature span of about 5.2 K.

## Discussion

In summary, the enormous E-CEs found in the TPEs were a consequence of the reversible conformational changes at orientation structure transition, which could occur in a *T*_a_ range higher than the *T*_g_ of the elastic segments. In particular, a uniform chain-length significantly improved the E-CEs of the TPEs during deformation. We anticipate that the enhanced E-CEs will also be encountered in other polymers with uniform chain-lengths. Furthermore, the generated heat was separated and conducted of the rotary-motion cooling device, which was appropriate to the requirement of high strains. Our work would inspire the study of E-CEs in polymer elastomers, as well as the development of solid-state cooling devices that could help combat climate change^4^.

## Methods

### Materials

Triblock poly(styrene-b-ethylene-co-butylene-b-styrene) (SEBS) thermoplastic elastomers (TPEs) used in this study was provided by KRATON Polymers Co., Ltd. (U.S.), Sinopec Group Co., Ltd. (China) and TSRC Industries Co., Ltd. (Nantong, China). The weight fraction of polystyrene (PS) of SEBS elastomer was evaluated as ~30 wt%. The molecular properties of these SEBS samples were assessed by GPC. The weight average molecular weights from sample TPEs-1 to sample TPEs-7 were 76,000 g mol^−1^, 156,000 g mol^−1^, 208,000 g mol^−1^, 308,000 g mol^−1^, 318,000 g mol^−1^, 350,000 g mol^−1^ and 414,000 g mol^−1^ respectively. Details of the molecular properties and sample labeling of these samples are illustrated in Supplementary Table 2, Supplementary Fig. 1 and Fig. 2e.

### Preparation

Sample sheets (0.4–1 mm thickness) were prepared by hot pressing process at 240 °C and 15 MPa for 20 m. Test specimens for two-dimensional wide-angle X-ray diffraction (2D SAXS), two-dimensional small-angle X-ray scattering (2D SAXS), Dynamic mechanical measurement (DMA) and mechanical characterization (ASTM D4482, active area is about 25 mm in length, 4 mm in width and 1 mm in thickness) and elastocaloric effects (E-CEs) measurement (active area is about 25 mm in length, 18 mm in width and 0.4 mm in thickness) were die cut from the same molded SEBS sheet. All samples were kept at the required ambient temperature for ten minutes before testing.

### Characterization

The Δ*T*_adi_ associated with the E-CEs in the TPEs was induced in an open indoor environment, heat sink and heat source in Fig. 1a are ambient air environment in this paper. The E-CE produced by the SEBS test specimens was carried out using a custom instrument, including stretching device and temperature measuring device (Supplementary Fig. 2). The stretching device comprised a precision ball screw and a linear guide rail, driven by an AC servo motor and controller (ECMA, Delta Electronics Co., LTD). Two ends of test specimen were mounted respectively on the endplate and the slide table of guide rail by jaw clamps. Servo motor drive the slide table to do precision repositioning movement on the linear guide rail, which cause the test specimen to undergo a controlled uniaxial strain. During the recovery process, the sider recovers its original position and the specimen contracts spontaneously. Sample strain is derived from the slider displacement. Direct measurement of Δ*T*_adi_ was performed by the examination of sample surface temperature (details in Supplementary Discussion 1). The surface temperature of deforming test specimen was measured by on-line infrared thermometer (ABSD-01A, Aobosaide Automation Technology Co., LTD; BRW600-406, Hunan Firstrate Sencer Co., Ltd) and visualized by an infrared thermal imager (T890-2, Testo SE & Co. KGaA). Temperature information detected by infrared thermometer was recorded at a frame rate of 9 s^−1^, and it was averaged on an area of ~0.5 cm^2^ at a fixed infrared emissivity of 0.91. The molecular properties of these TPEs were assessed by a gel permeation chromatography (GPC, PL-GPC220, Agilent technologies, CA, USA). The GPC measurements were carried out at 40 °C using THF as the eluent at a flow rate of 1 mL min^−1^. Transmission electron microscopy (TEM) images were obtained on FEI Tecnai G2 F30 with an accelerating voltage of 300 kV. The samples were ultramicrotomed at −130 °C to a section with a thickness of about 70 nm. The sections were then stained with 0.5 wt% RuO_4_ vapor at 50 °C for 30 min in order to stain selectively polystyrene (PS) domains. RuO_4_ is a highly toxic vapor, please use it carefully. The structure evolution of SEBS samples was in situ monitored by 2D WAXS and SAXS, which were performed using Genix 3D X beamline with wavelength λ = 1.54 Å at Xeuss 2.0 (Xenocs). The sample-to-detector distances for SAXS and WAXS were set to be 2497.7 and 151.7 mm, respectively. The one-dimensional SAXS profiles were acquired by the circularly averaging of the intensity with the build-in software. The value of the scattering wave vector magnitude is given by $q=\frac{4\pi \,\sin \,\theta }{\lambda }$ where 2*θ* is the scattering angle. Mechanical tests were run in an electromechanical universal testing machine (E44.104, MTS systems Co., LTD) equipped with a BSA-XS-50kg force transducer and a CEC1200 temperature testing chamber. DMA measurement of SEBS samples was carried out on a dynamic mechanical analyzer (DMA, PE-DMA8000) at 1 Hz with a heating rate of 2 K min^−1^ in the temperature range of 173–443 K. The specific heat capacity (*c*_*p*_) was confirmed by differential scanning calorimetry (DSC, TA-DSC2500) measurement with a heating rate of 10 K min^−1^ in the temperature range of 203–443 K.

## Supplementary information

Supplementary Information

## Data Availability

The data that support the findings of this study are available from the corresponding author upon reasonable request.

## References

[1] Klinar K, Kitanovski A (2020). Thermal control elements for caloric energy conversion. Renew. Sust. Energ. Rev..

[2] Cazorla C (2019). Novel mechanocaloric materials for solid-state cooling applications. Phys. Rev. Appl..

[3] Moya X, Kar-Narayan S, Mathur ND (2014). Caloric materials near ferroic phase transitions. Nat. Mater..

[4] Li T (2019). A radiative cooling structural material. Science.

[5] Porenta L (2020). Thin-walled Ni-Ti tubes under compression ideal candidates for efficient and fatigue-resistant elastocaloric cooling. Appl. Mater. Today.

[6] Ouyang GY (2020). Elastocaloric effect in vanadium (IV) oxide. Appl. Phys. Lett..

[7] Manosa L, Planes A (2017). Materials with giant mechanocaloric effects: cooling by strength. Adv. Mater..

[8] Li B (2019). Colossal barocaloric effects in plastic crystals. Nature.

[9] Joule JP (1857). On some thermo-dynamic properties of solids. Philos. Trans..

[10] Kitanovski A (2020). Energy applications of magnetocaloric materials. Adv. Energy Mater..

[11] Neese B (2008). Large electrocaloric effect in ferroelectric polymers near room temperature. Science.

[12] Tegus O, Brück E, Buschow KH, de Boer FR (2002). Transition-metal-based magnetic refrigerants for room-temperature applications. Nature.

[13] Ma RJ (2017). Highly efficient electrocaloric cooling with electrostatic actuation. Science.

[14] Wang R (2019). Torsional refrigeration by twisted, coiled, and supercoiled fibers. Science.

[15] Manosa L (2010). Giant solid-state barocaloric effect in the Ni-Mn-In magnetic shape-memory alloy. Nat. Mater..

[16] Ulpiani G (2020). Upscaling of SMA film-based elastocaloric cooling. Appl. Therm. Eng..

[17] Cong DY (2019). Colossal elastocaloric effect in ferroelastic Ni-Mn-Ti alloys. Phys. Rev. Lett..

[18] Moya X, Mathur ND (2020). Caloric materials for cooling and heating. Science.

[19] Greibich F (2021). Elastocaloric heat pump with specific cooling power of 20.9 W g–1 exploiting snap-through instability and strain-induced crystallization. Nat. Energy.

[20] Tusek J (2016). A regenerative elastocaloric heat pump. Nat. Energy.

[21] Hou HL (2019). Fatigue-resistant high-performance elastocaloric materials made by additive manufacturing. Science.

[22] Qian SX, Wang Y, Yuan LF, Yu JL (2019). A heat driven elastocaloric cooling system. Energy.

[23] Qian SX, Yuan LF, Yu JL, Yan G (2017). Numerical modeling of an active elastocaloric regenerator refrigerator with phase transformation kinetics and the matching principle for materials selection. Energy.

[24] Tusek J (2015). The elastocaloric effect: a way to cool efficiently. Adv. Energy Mater..

[25] Xie ZJ, Wei C, Guyomar D, Sebald G (2016). Validity of Flory’s model for describing equilibrium strain-induced crystallization (SIC) and thermal behavior in natural rubber. Polymer.

[26] Guyomar D (2013). Elastocaloric modeling of natural rubber. Appl. Therm. Eng..

[27] Xie ZJ, Sebald G, Guyomar D (2016). Comparison of direct and indirect measurement of the elastocaloric effect in natural rubber. Appl. Phys. Lett..

[28] Patel S, Chauhan A, Vaish R, Thomas P (2016). Elastocaloric and barocaloric effects in polyvinylidene di-fluoride-based polymers. Appl. Phys. Lett..

[29] Yoshida Y, Yuse K, Guyomar D, Capsal JF, Sebald G (2016). Elastocaloric effect in poly(vinylidene fluoride-trifluoroethylene-chlorotrifluoroethylene) terpolymer. Appl. Phys. Lett..

[30] Bom NM, Imamura W, Usuda EO, Paixao LS, Carvalho AMG (2017). Giant barocaloric effects in natural rubber: a relevant step toward solid-state cooling. ACS Macro Lett..

