# Supplementary material for: Solid-state cooling by elastocaloric polymer with uniform chain-lengths
Source: Nat Commun. 2022 Jan 10;13:9. doi: 10.1038/s41467-021-27746-y (PMC8748545; doi:10.1038/s41467-021-27746-y)
Supplement: Supplementary file 1 — Supplementary Information [file 41467_2021_27746_MOESM1_ESM.pdf]

Supplementary Information for

Solid-state cooling by elastocaloric polymer with uniform chain-lengths

Shixian Zhang[1]†, Quanling Yang[1]†, Chenjian Li[1], Yuheng Fu[1], Huaqing Zhang[1], Zhiwei Ye[1], Xingnan Zhou[1], Qi Li[2], Tao Wang[1], Shan Wang[1], Wenqing Zhang[3], Chuanxi Xiong[1]*, Qing Wang[4]*

Corresponding authors. Email: cxiong@whut.edu.cn (Chuanxi Xiong[1]); wang@matse.psu.edu (Qing Wang[4]*).

†Shixian Zhang and Quanling Yang contributed equally to this work.

This Supplementary Information file includes:

Supplementary Discussions 1 to 7

Supplementary Figs. 1 to 38

Supplementary Table 1 to 4

Supplementary References 1 to 35

Supplementary Discussions

Supplementary Discussion 1. Determination of the adiabatic strain rate.

The adiabatic temperature variations ($|\Delta T_{adi}|$) of TPEs during their recovery processes (from 600% strain to 0% strain) were first tested at different strain rates in a vacuum test chamber (the absolute pressure was about 10^4 Pa) by using a thermal couple (K-type). The vacuum test chamber can avoid convection heat transfer and simulate the adiabatic environment. As illustrated in Supplementary Fig. 3, the temperature variation of all samples tested at different strain rates remain constant, testifying that the intrinsic E-CE of the samples is independent on the strain rate.

Direct measurement of $|\Delta T|$ values of TPEs during the cooling process (process 3-4 in Fig. 1a) was further performed by the examination of sample surface temperature in an open indoor environment via a non-contact infrared thermometry. Supplementary Fig. 4 reveals a sharp increase in $|\Delta T|$ value within the 0.3–3 s^{-1} strain rate range, which indicated that the $|\Delta T|$ values measured in the open indoor environment is related to the strain rate. This is caused by the convection and radiation heat transfer between the test sample and ambient environment at low strain rates. Due to the application time of external force is shortened in the stretching process, the heat transfer between the test sample and ambient environment decreases significantly with the increasing of strain rate. When the strain rate is greater than 5 s^{-1}, the $|\Delta T|$ values of TPEs remain constant and are equivalent to the $|\Delta T_{adi}|$ values measured in the vacuum test chamber. Therefore, it can be considered that the measurement at a high strain rate (15 s^{-1}) in the open indoor environment is equivalent to the adiabatic test condition. It should be noted that the conductive heat loss also occurs when the sample is in contact with the fixing jaws. However, as illustrated in the infrared images (Fig. 1c and Supplementary Fig. 19), this conductive heat loss is negligible.

Although the temperature test results are more accurate by using the vacuum test chamber even at low strain rate, the quasi-vacuum environment is disadvantageous to the heat exchange processes of the E-CE cycles. In comparison, the open indoor environment testing is more convenient. Considering the requirement for the long-lasting measurements in this work, the examination conditions with a strain rate of 15 s^{-1} in an open indoor environment were selected for quantifying ΔT_{adi}.

Supplementary Discussion 2. Aggregate structure evolution during elastocaloric cycles.

In order to further explain the energy conversion relationship of the E-CE in the SEBS system, in-suit SAXS/WAXD, TEM and mechanical tests were utilized in combination to monitor the aggregate structure evolution of TPEs-1 at different strain level.

As shown in Supplementary Fig. 7, the evolution of SEBS aggregate structure is illustrated by the sequential procedures of stretching. Supplementary Fig. 8 shows selected 2D SAXS patterns of TPEs-1 at different strain levels deformed at room temperature. The 2D SAXS pattern of unstretched sample is an isotropic circular symmetrical scattering pattern, which indicates a statistically random spherically symmetric microparticle aggregate structure of PS domain morphology. Fig. 2a shows TEM images for TPEs-1 at initial state. PS cylinders (black area) are randomly distributed in the PE/PB matrix in three dimensions. And the SEBS has the hexagonal-packed cylinders vertical to the paper plane. PE/PB segments and PS segments are linked by covalent bond, so that the external force can transfer between these adjacent PS and PE/PB domains. Therefore, when the hard PS phase is randomly distributed, the PE/PB soft segments undergo no tensile force and has random conformation. The TEM results are consistent with those observed from SAXS.

Supplementary Fig. 9 shows the 1D SAXS profiles obtained by full tilted circular integration of corresponding 2D SAXS patterns (Supplementary Fig. 8). From top to bottom, it reflects the average intensity (I) as a function of scattering wave vector (q) for different strain levels. Among them, the bottom profile is the 1D SAXS corresponding to the recovery of strain. At strain 0%, the relative peak positions of multiple Bragg reflections provide the fingerprints of ordered PS grains. TPEs-1 in the original state has a first-order peak 1(q*) = 0.0232 $Å^{-1}$. The arrows representing the locations of allowed Bragg reflections based on the firs-order peak position. Relative q-value of these reflections at 1q*, $\sqrt{3}$ q*, 2q*, $\sqrt{7}$ q*, 3q* clearly indicating the hexagonal packed microstructure of PS cylindrical microdomains (HPC)[1]. The internal structure of PS domains is amorphous at the long-range structure level.

At the strain of 5%, the first-order particle scattering ring of 2D SAXS pattern deforms from a circle shape (strain of 0%) into an ellipse one, which indicates a statistically ellipsoidal symmetric microparticle aggregate structure of PS domain morphology. The deformed HPC structure has an ellipsoidal symmetric orientation. The corresponding 1D SAXS shows a principal scattering wave vector position of 1(q*) = 0.0227 $Å^{-1}$, followed by a q-value ratio of 1q*:$\sqrt{3}$q*: $\sqrt{7}$q*, indicating that the HPC microstructures of PS domains are still preserved. Compared with unstretched 1D SAXS profile, the primary scattering wave vector positions under 5% strain move slightly to a lower q-value, which is caused by the increase of d-space between PS cylinders under tensile stress. And this results in the stretching of the PE/PB soft segments between adjacent PS cylinders.

Supplementary Fig. 10 shows the stress-strain curve of TPEs-1 undergo the first stretching recovery cycle. It can be considered that the deformation in the initial linear elastic region (strain of 0% – 7%) in the stress-strain curve is mainly caused by the elongation of elastic soft segments between PS cylinders. When the strain continues to increase, the stress-strain curve exhibits yield

behavior followed by a stress relaxation effect, and the modulus of TPEs-1 decrease significantly. This phenomenon may be related to the untangling of PE/PB soft segments and the adjustment of spatial position of PS cylinders.

From the 2D SAXS pattern with a strain of 50%, the ellipse scattering ring transforms into four bright arcs, and the concave surfaces of the four scattering spots are centered along the equator. Similar to the ellipse pattern, it can be considered as a slightly deformed elliptical symmetric structure. PS cylinders with elliptical symmetric structure undergo rotation and slip along the stretching direction in the further stretching process. The rotated and shuffling cylinders are preferentially distributed in two symmetrical directions resulting in the four-point scattering pattern. In the corresponding 1D SAXS profile at 50% strain, the full-width at half maximum (FWHM) of primary Bragg reflection peak increases gradually, which indicates the decreasing in the size of HPC grain. Rotated and shuffling PS cylinders is continuously stripped from the HPC microstructures due to the stretching of chemical bond connected soft segments. Correspondingly, the rotated PS cylinders can continuously curtail the elongation ratio of oriented PE/PB soft segments to offset the stress, which resulting the stress relaxation effect.

When the strain is greater than 200%, the stress-strain curve gradually demonstrates a stress enhancement effect, which is related to the ultimate elongation of the soft segments. From the 2D SAXS pattern at a strain of 200%, it can be seen that the four-point scattering pattern straightens and moves to a relative low q-value. This indicates the d-space of PS cylinders have further increased and the HPC microstructures have been completely destroyed. At the same time, PS cylinders are further oriented in the stretching direction. When the strain increases to 600%, clear vertical stripes can be manifest from the 2D SAXS pattern, which indicates a small angle between PS cylinder and the tensile direction. The PS cylinders can form a statistically mirror-symmetric

arrangement along the stretching direction. In the 1D SAXS profiles with a strain of 300% – 600%, the Bragg reflection disappears completely. And a shoulder peak appears near q=0.03 $Å^{-1}$ of which position remains constant with strain. This diffraction corresponds to the shape factor scattering of single cylinder particles[2, 3]. The PS cylinders and PE/PB soft segments are fully orientated along the stretching direction.

When the strain returns to the initial state, the 2D SAXS basically returns to the circular pattern. The 1D SAXS consists of a series of Bragg reflections with q-value ratio of 1q*: $\sqrt{3}$q*: 2q*: $\sqrt{7}$q*: 3q*, which proves that the PS hard segments return to the disorderly distributed HPC microstructure. Accordingly, the oriented PE/PB soft segment reverts to the state of random coils.

Fig. 2b and Supplementary Fig. 6 show the 2D WAXD pattern of TPEs-1 under different strain levels. While the relative intensity of the original diffraction ring along the stretching direction is sacrificed, the bright arc perpendicular to the stretching direction is gradually concentrated. And this change is more obvious in the strain range of 200%–600%. This can also prove the stress relaxation effect at a strain of 0%–200% as well as the stress enhancement effect at a strain of 200%–600%. Under the strain of 0%–200%, the rotated and shuffling PS cylinders can provide more free-space for oriented soft molecular chains, which reduces the tendency of chain orientation. In the stress enhancement stage where the strain is 200%–600%, the HPC microstructures are completely destroyed into fully oriented single PS cylinders, the degree of molecular chain (PE/PB segments) orientation will increase rapidly under the drive of an external field. But even at a strain of 600%, there is no crystallization signal appeared, and the anisotropic amorphous ring is found to persist throughout the stretching process. The WAXD diffraction patterns after deformation is considered as the superposition of the oriented molecular chain diffraction pattern and the residual amorphous halo.

In summary, during the stretching and recovery process of SEBS, the application and removal of stress mainly lead to the orientation and recovery of the soft segment, as well as the rotation and slip of the PS microstructure which driven by the soft segment. Compared with the change in the orientation of the soft molecular chain, the energy consumed by the rotation and deformation of the PS domains and the entire cylinders can be neglected.

Supplementary Discussion 3. Theoretical derivation of elastocalric ΔS_{iso} and ΔT_{adi} in SEBS thermoplastic elastomer driven by conformational change

Direct measurement of ΔS_{iso} related to E-CEs is still a challenge. Direct or quasi-direct measurements of ΔS_{iso} is mostly reported in the researches of electrocaloric effect, magnetocaloric effect and barocaloric up to now[4-8]. This problem can be partially solved by an indirect calculation of ΔS_{iso} with the Maxwell relation $(\frac{\partial S}{\partial \varepsilon})_T = V_0(\frac{\partial \sigma}{\partial T})_\varepsilon$. For the isothermal change process, corresponding expression for ΔS_{iso} is given by:

$$\Delta S_{iso} = V_0 \int_0^{\varepsilon} \left(\frac{\partial \sigma}{\partial T}\right)_{\varepsilon} \mathrm{d}\varepsilon \qquad (1)$$

Where, T is the temperature (K), V_0 is the original volume of specimen and is assumed to be constant, σ is the engineering stress and ε is the engineering strain.

The ΔS_{iso} derived from the Maxwell relation as expressed in Supplementary Equation (1) renders the Clausius-Clapeyron equation. The validity of these equations is based on the assumption that the thermodynamic system is ergodic. Regarding polymer materials, some elastomers can undergo strain induced crystallization (SIC), i.e., first order phase transition; however, these transitions are spread over a relatively large range of strain or stress, which lead to an unclear definition of the stress or strain interval corresponding to the phase transition process, and further reflect the errors in the calculation results[9]. This may be caused by the complex non-

ergodic behavior (e.g. conformational adjustment) of polymer materials during their SIC processes. This is at variance to the traditional entropic elasticity theory and rubber elasticity statistical theory which describes the relationship among the molecular conformation and macroscopic physical quantities in the process of elastomer deformation[10].

According to the traditional entropic elasticity theory, the internal energy of the elastomer system is constant in the process of deformation cycle, and the contribution of latent heat (corresponding to the first-order phase transition) is ignored. During the adiabatic stretching process, the applied stress simply changes the conformation (ΔS_λ) of elastomer molecular chain from the original crimp state to the extended state, so that external work ($dW = f dl$) can convert into sensible heat, i.e, $dW = -T dS_\lambda > 0$ ($dW < 0$, when the system is doing work). Therefore, ΔT_{adi} and ΔS_{iso} can be deduced from the change of conformational entropy:

$$\Delta S_\lambda = -mc_p \ln\frac{T_2}{T_a} \tag{2}$$

$$T_2 = T_a \exp\left(\frac{-\Delta S_\lambda}{mc_p}\right) \tag{3}$$

where, m is the sample mass; c_p is the specific heat. Considering that near room temperature, c_p tend to be one order of magnitude larger than ΔS_λ. Using the Taylor's development of the exponential function, a new equation is obtained:

$$T_2 \approx T_a - T_a\frac{\Delta S_\lambda}{mc_p} \tag{4}$$

For uniaxial tension, conformational entropy change ΔS_λ can be further quantified by the statistical theory of rubber elasticity:

$$\Delta S_\lambda = -\frac{1}{2}Nk\left(\lambda^2 + \frac{2}{\lambda} - 3\right) \tag{5}$$

where, N is the network chain density; k is the Boltzmann's constant and $\lambda = \frac{l - l_{0\text{-PS}}}{l_{0\text{-PE/PB}}}$ is the principal elongation ratio. l is the sample length, l_0 is the initial length of the sample, $l_{0\text{-PS}}$ is the initial length of PS phase, $l_{0\text{-PE/PB}}$ is the initial length of PE/PB phase. $l_{0\text{-PS}}$ remains unchanged during sample deformation, and it can be approximately be calculated by $l_{0\text{-PS}} \approx \frac{30}{70} \times \frac{\rho_{\text{PE/PB}} l_{0\text{-PE/PB}}}{\rho_{\text{PS}}} = 0.28\ l_0$. Approximately, $\lambda \approx \frac{\varepsilon + 1}{0.72} - 0.39$.

Supplementary Equation (5) is established under the assumption of affine deformation. However, the deformation of the experimental crosslinked network is not completely affine deformation, especially when the chain-length is uneven. Generally, the non-affine deformation will reduce the network chain density (N). As a simple correction, a correction factor A_φ can be introduced into N[11]. And a new equation is given:

$$\Delta S_\lambda = -\frac{1}{2} A_\varphi N k \left(\lambda^2 + \frac{2}{\lambda} - 3\right) \tag{6}$$

where, $0 \leq A_\varphi \leq 1$. When the chain-length is completely uniform (MR = 0), $A_\varphi = 1$. When MR trends to infinity, $A_\varphi = 0$.

Based on Supplementary Equation (4) and (6), ΔT_{adi} is obtained as follow:

$$\Delta T_{\text{adi}} = T_2 - T_{\text{a}} = \frac{1}{2} A_\varphi N k T_{\text{a}} \left(\lambda^2 + \frac{2}{\lambda} - 3\right)\left(\frac{1}{m c_{\text{p}}}\right)$$

$$= \frac{1}{2} A_\varphi N k T_a \left(\lambda^2 + \frac{2}{\lambda} - 3\right)\left(\frac{1}{\rho V_0 c_p}\right) \tag{7}$$

For solid matter under small deformation, $\frac{A_\varphi N k T_{\text{a}}}{V_0} = \frac{E}{3}$, where E is the Young's modulus; V_0 is the initial sample volume. A new equation for ΔT_{adi} is obtained as follow:

$$\Delta T_{\text{adi}} = \frac{1}{6} E \left(\lambda^2 + \frac{2}{\lambda} - 3\right)\left(\frac{1}{\rho c_{\text{p}}}\right) \tag{8}$$

In the isothermal process (b−c), the applied strain remains constant, and the system reduces its temperature by releasing sensible heat until the thermal equilibrium is reached. Therefore, the change of thermal entropy during heat exchange process is as follow:

$$\Delta S_{\mathrm{iso}} = \frac{1}{m}\int_{T_2}^{T_\mathrm{a}} \frac{1}{T}\mathrm{d}Q = \int_{T_2}^{T_\mathrm{a}} \frac{c_\mathrm{p}}{T}\mathrm{d}T \tag{9}$$

Accordingly, after the removing of external field, its inverse process, namely, the retraction process of the deformed elastomer can be spontaneous: System work to reduce its temperature, then absorbs heat from the environment and finally returns to the initial state. Similarly, ΔT_{adi}, ΔS_{λ} and ΔS_{iso} on cooling process can be deduced as follow:

$$\Delta T_{\mathrm{adi}} = -\ \frac{1}{6}E\left(\lambda^2 + \frac{2}{\lambda}\ -\ 3\right)\left(\frac{1}{\rho c_\mathrm{p}}\right) \tag{10}$$

$$\Delta S_{\lambda} = \frac{1}{2}A_{\varphi}Nk\left(\lambda^2 + \frac{2}{\lambda} - 3\right) \tag{11}$$

$$\Delta S_{\mathrm{iso}} = \frac{1}{m}\int_{T_4}^{T_\mathrm{a}} \frac{1}{T}\mathrm{d}Q = \int_{T_4}^{T_\mathrm{a}} \frac{c_\mathrm{p}}{T}\mathrm{d}T \tag{12}$$

Supplementary Fig. 11a to Supplementary Fig. 17a show the stress-strain curves of TPEs during the first ten stretch recovery cycles. The black arrows point to the increasing number of cycles. When TPEs undergo their first stretching cycle, there are physical entanglements and interlocking structures in the local areas of the original random PE/PB soft segments and the randomly distributed PS domains. In the process of adjusting the molecular conformation through an external field, these rigid entanglement points and interlocking structures can contribute significant internal stress. But this part of stress contribution is irrecoverable. After 2–10 cycle, the stress-strain curves almost coincide which indicates the irreversible contribution to the modulus can be eliminated. Therefore, the sample modulus of E can be obtained by calculating the slope of the initial elastic region of the stress-strain curve after multiple stretch recovery cycles

(Supplementary Table 3). The specific gravity (ρ) of TPEs were determined as 0.91 g cm^{-3} by the method of ISO2781 test.

When Supplementary Equation (5) is used to deduction the $|\Delta T_{adi}|$, it will lead to an error in the theoretical value. It can be considered that this difference is caused by the inaccurate consideration of the multiple phase situation in Supplementary Equation (5). At small strain region, the effect of the length difference of molecular chains on the conformation change is weak. But when the deformation is increasing, the size limitation effect of the short chains becomes more prominent, which leads to the impossibility of conformation change of the long chains (ideal model in Supplementary Figs. 11c, 11d). So that the temperature is lower in the experimental measurement of temperature change. On the other hand, theoretical $|\Delta T_{adi}|$ can be calculated by Supplementary Equation (10) (derived from Supplementary Equation (6)). Supplementary Fig. 11b to Supplementary Fig. 17b show the strain evolution of the theoretical and experimental $|\Delta T_{adi}|$ in the cooling process. In the strain interval of 0%–100%, theoretical $|\Delta T_{adi}|$ almost agrees with the experimental results. Under the same strain level where the strain is greater than 100%, the test temperature change is lower than the theoretical calculation value. When the strain ε>300%, this temperature difference is more significant. It can be considered that the assumption of Gaussian chain is no longer valid at a high strain, and the external force needs to input more work to change the conformation of molecular chains[12, 13]. The source of entropy changes decreases under unit strain, so that it brings lower conformational entropy change under unit strain. These lead to the E-CEs ΔS_{iso} derived from Supplementary Equation (12) smaller than that derived from Supplementary Equation (11). Accordingly, these reasons lead to a smaller measured value of $|\Delta T_{adi}|$ than the calculated value of Supplementary Equation (10).

Supplementary Discussion 4. Ambient temperature dependence of E-CEs in TPEs-1

As shown in Supplementary Fig. 20. When T_a rises above the T_g of elastic soft segments (S-T_g), the soft segments in rubber state has obtained enough energy to rotate freely. According to the principle of entropy increase, freely rotating soft segments have a tendency in the curled state to obtain the maximum conformational entropy. "*E*" represents the property of polymers to resist tensile force and maintain the curled state of molecular chains. Therefore, the tendency of molecular chain curling is intensified with the rising of T_a and is manifested as an increase in *E*. This means that the external force needs to input more work for the conformational transformation of TPEs-1 at a higher T_a, resulting in a greater ΔS_{iso} and ΔT_{adi}.

Supplementary Discussion 5. Derivation of COP in TPEs

The COP_{mat} for TPEs-1 near room temperature is described by the ratio of the cooling energy per unit mass (Q/m) to the input work per unit mass ($\Delta W/m$):

$$\mathrm{COP_{mat}} = \frac{\frac{Q}{m}}{\frac{\Delta W}{m}} = \frac{\int_{T_4}^{T_a} c_p \mathrm{d}T}{\frac{1}{m}\oint F\mathrm{d}l} = \frac{\int_{T_4}^{T_a} c_p \mathrm{d}T}{\frac{1}{\rho A_0 l_0}\oint F\mathrm{d}l} = \frac{\int_{T_4}^{T_a} c_p \mathrm{d}T}{\frac{1}{\rho l_0}\oint \sigma\mathrm{d}l} \quad (13)$$

where, F is the tensile force, ρ is the sample density, V_0 is the sample volume, l_0 is the initial sample length, l is the sample length and A_0 is the initial cross section area of the sample. The hysteresis of stress-displacement as well as ΔT_{adi} on heating and cooling process (Supplementary Fig. 21 to Supplementary Fig. 27, Supplementary Fig. 29 to Supplementary Fig. 35) gradually stabilized after the first 20 cycles. $\Delta W/m = \frac{1}{\rho l_0}\oint \sigma\mathrm{d}l$, where $\oint \sigma\mathrm{d}l$ is calculated from the hysteresis of stress-displacement characteristics as displayed in Supplementary Fig. 21 to Supplementary Fig. 27 by integrating stress (σ) over displacement (l) for stretching and recovery cycles. Q/m =

$\int_{T_4}^{T_a} c_p \mathrm{d}T$ can be calculated by integrating area of specific heat capacity curve from T_4 to T_a in Fig. 2c and Supplementary Fig. 28.

Supplementary Discussion 6. Cyclic behaviors of E-CE for TPEs

The cyclic behaviors of the E-CE for TPEs are shown in Supplementary Fig 29. to Supplementary Fig 35. All samples were tested at room temperature with a strain rate of 15 s^{-1}. TPEs exhibit stable ΔT_{adi}, Q/m, $\Delta W/m$ and COP_{mat} values during their 40 times of cycles, explaining its steady cycling performance. The COP_{mat} values of TPEs decreases from 17.9 to 8.5 with the increasing of MR. It can be considered that for TPEs with nonuniform chain-length, the over-strained polymer chains should induce addition mechanical losses, which leads to the decrease of COP_{mat} value.

On the other hand, due to the inherent viscoelasticity of polymer materials, the fatigue life of TPEs is generally low at a very high strain rate (15 s^{-1}). At the same time, due to the inevitable defects in the process of sample preparation, the fatigue life of all samples tested under a strain rate of 15 s^{-1} was over 40 cycles. When polymers deform at a very high strain rate, the movement of the local molecular chains is out of sync with the deformations, which will lead to the local strain or stress in the polymer exceeding its fracture strain or ultimate stress, and finally lead to the fracture of the sample. This will also induce addition mechanical losses during the E-CE cycles. But the fatigue life can be remarkably improved at a lower strain rate. The fatigue life of TPEs-1 samples tested under a strain rate of 1 s^{-1} was over 1000 cycles.

In order to avoid the reduction of fatigue life caused by local overload in the high-speed extension process, we further tested the cyclic behavior of TPEs-1 by means of low-speed extension and high-speed retraction. The strain rates were set as 1 s^{-1} in the extension process and 15 s^{-1} in the retraction process. As shown in Supplementary Fig. 36, the ΔT value of the extension

process is about 12 K at the beginning of the cycles, which is lower than that in the high-speed extension test. It can be considered that the time required for low-speed extension process was relatively long, which led to the convection heat transfer between the test sample and environment. During the high-speed retraction process, the $|\Delta T_{adi}|$ value was about 15 K, which is the same as that in Fig. 1b. Furthermore, TPEs-1 exhibit a stable $|\Delta T_{adi}|$ change trend during 1000 times of E-CE cycles. According to the fitted regression lines, compared with the first E-CE cycle, the $|\Delta T_{adi}|$ value retention rate of the cooling process after 1000 times of E-CE cycles reached 96%, which shows that TPEs-1 exhibited steady cycling performance.

As the working medium of solid-state cooling device, improving the fatigue life of TPEs is an issue worthy of further exploration. Some studies have also found that reducing the strain rate can significantly improve the fatigue life[14]. By replacing the current die cut sample preparation process with injection molding processing, the defects and burrs in the die cut process can be significantly reduced, so as to improve the fatigue life. The fatigue properties of polymers can also be improved by adding appropriate additives[15]. Furthermore, the fatigue damage of SEBS can be repaired by high temperature due to its thermoplasticity, which is also the advantage of using this kind of materials.

Supplementary Discussion 7. Rotary motion elasto-based cooling device based on TPEs-1

The linear motion uniaxial tension system (Supplementary Fig. 2) requires a large length of the equipment (7 times of sample length when the strain is 600%) to drive the E-CE of polymers. The large sample deformation is disadvantaged to the design of cooling device and the export of cooling energy. In view of this, the cooling device (about 2.5 times of sample length when the strain is 600%) illustrated in Supplementary Fig. 38 and Fig. 4a was used for heat separation and

discharge by a stream of water. A precision pump (YZ1515X-A, Longer Pump Co., Ltd.) was used for pumping the water at a constant flow rate of 1.3 ml s^{-1}. Two thermal couples (K-type) were used to test inlet and outlet water temperature.

The cooling device unit consists of cover plate (U3 in Supplementary Fig. 38a), body case (U1 in Supplementary Fig. 38b and Fig.4a) and revolving disc (M in Supplementary Fig. 38c and Fig. 4a) was made of polytetrafluoroethylene (PTFE). 2 mm gap (U2 in Fig.4b) was designed between the outer wall of revolving disc and the inner wall of the body case groove. A 2 mm thick cavity (U2 in Fig.4b) could be formed after sealing by the cover plate, which allowed TPEs-1 sample and water to move freely. The effective area of TPEs-1 (S1 in Supplementary Fig. 38c and Fig. 4a) was about 30 mm in length, 18 mm in width and 1 mm in thickness. Two ends of the sample were fixed by two clips (Green part in Supplementary Fig. 38c and Fig. 4a) on the body case and the revolving disc respectively, so that the revolving disc could drive the elongation and recovery of TPEs-1. M1 (red part) in Supplementary Fig. 38c were a series of cylinders which fixed on the periphery of the revolving disc that can move independently to reduce friction and ensure uniform stretching of TPEs-1. The symbol Water in Supplementary Figs. 38b and Fig. 4a represented the storage water in the cavity (U2), and the mass of the storage water during elastocaloric cycles was 4.9 times than that of the effective part of TPEs-1. The mass of storage water remains constant during elastocaloric cycles. By increasing the width of the current sample (3.0 cm in length, 1.8 cm in width and 0.1 cm in thickness) and duplicating the current cooling device, the overall size (including U2) of the cooling device with 10 W of cooling power could be obtained. The TPEs-1 sample for the upscale cooling device (10 W of cooling power) would have a mass of 5.2 g (3.0 cm in length, 19.3 cm in width and 0.1 cm in thickness). When the thickness

of the cavity U2 is 0.2 cm, the volume of the cooling device is about 764.1 cm^3 (the diameter is about 7.1 cm and the depth is about 19.3 cm).

During the cooling process, when the strain of 600% is fully recovered, the TPEs-1 specimen was completely immersed in storage water, which reduced the outlet water temperature by 1.1 K. According to $Q_{out} = m_{out}c_{p,out}\Delta T_{out}$, where Q_{out} is the cooling energy discharged by storage water, m_{out} is the mass of storage water, $c_{p,out}$ is the specific heat of storage water, ΔT_{out} is the temperature variation of out let water. The m_{out} can be estimated as 2.4 g. The $c_{p,out}$ can be estimated as 4.2 J g^{-1} K^{-1}. So that the water current can bring out about Q_{out} = 11.1 J of cooling energy for a single cooling cycle. The COP_{sys} and SCP_{sys} for the cooling process of the full system are defined by

$$COP_{sys} = \frac{Q_{out}}{W_{in}} = \frac{Q_{out}}{P_{in}t} \tag{14}$$

$$SCP_{sys} = \frac{Q_{out}}{mt} \tag{15}$$

where, Q_{out} is the cooling energy discharged by storage water, W_{in} is the input energy of the system during the recovery process, P_{in} is the input power of the system during the recovery process, t is the time required for the recovery process and m is the sample mass. The P_{in} value was evaluated as 0.1 W, which was tested by a power measurement socket (SPK-10A, Zhejiang Sicoele Technology Co., Ltd.). The t value was set as 12 s from the system (at an angular velocity of 0.52 rad s^{-1}). According to Supplementary Equation (14), the COP_{sys} can be estimated as 9.3.

During the heating process, ΔT_{out} of outlet water is relatively small (0.4 K) (Fig. 4b). This is mainly due to the fact that only part of the stretched sample underwent direct heat transfer with the bottom reservoir, and the heat of the rest part of the sample sheet was carried out to the bottom reservoir by current water. This will result in a low heat transfer value per unit time. By extending the water supply time, that is, increasing the mass of cooling water current to 3 times of the mass

of storage water, the water current took away about 12.1 J of the heat. The heat transfer time can be shortened by increasing the water flow rate at the heating end.

Furthermore, the cold exchanger (E1) was replaced by a heat storage tank and the silicone rubber tube was wrapping with polyethylene foam cotton to reduce the cold loss. This replacement can effectively recover cold energy form previous cycles and further reduce the water temperature after multiple refrigeration cycles. Under this replacement, the temperature change of the outlet water gradually stabilized to approximately −5.2 K after 10th cooling cycles. This indicated that the maximum temperature span of the system was about 5.2 K.

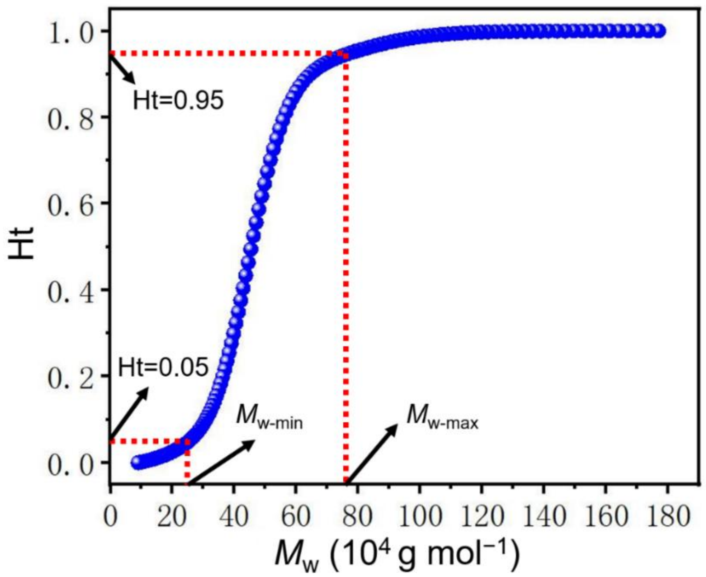

Supplementary Figure 1. GPC curves of TPEs-7. The maximum $M_{w\text{-}max}$ appears at Ht=0.95.The minimum $M_{w\text{-}min}$ appears at Ht=0.05.

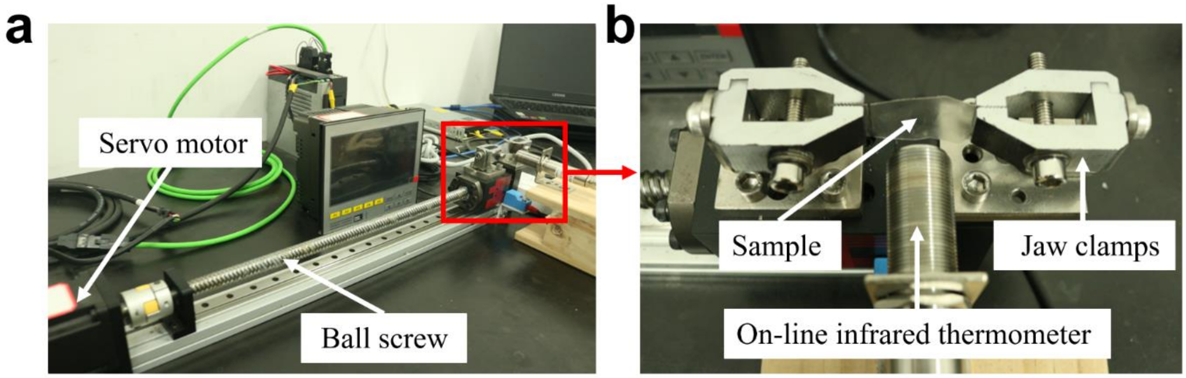

Supplementary Figure 2. a The uniaxial tension system in an indoor open environment for the directly quantification of elastocaloric ΔT_{adi}. **b** An amplicication in the red region of Supplementary Fig. 2a.

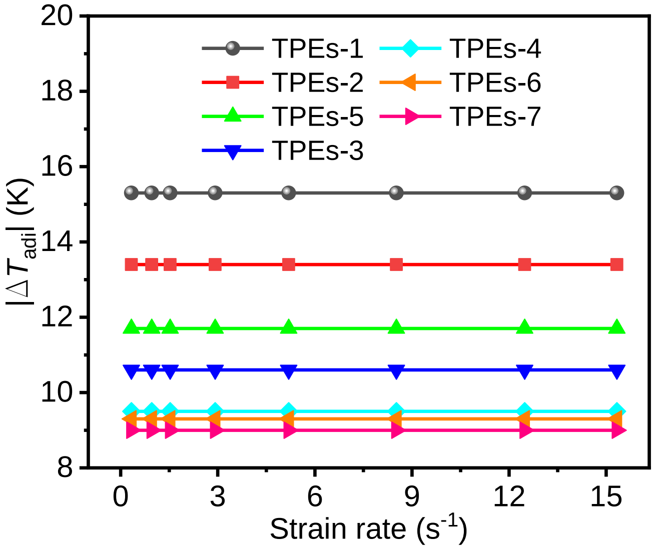

Supplementary Figure 3. $|\Delta T_{adi}|$ versus strain rate of TPEs at a constant strain of 600% during their recovery processes. All samples were measured in the vacuum test chamber.

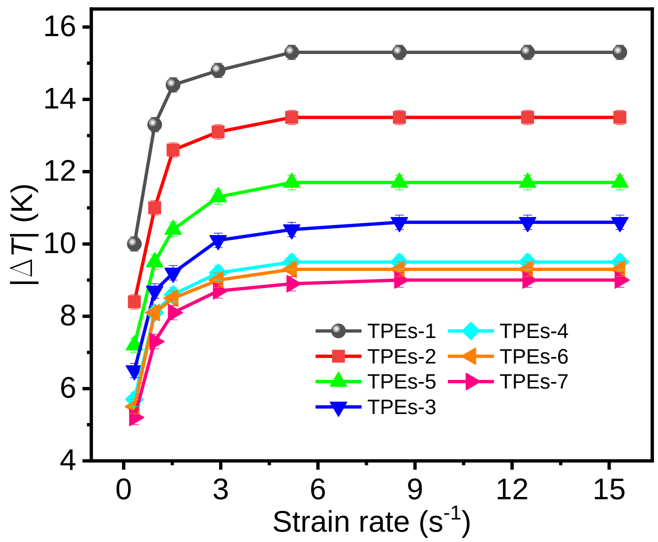

Supplementary Figure 4. Surface $|\Delta T|$ of TPEs as a function of strain rate. All samples were measured on elastocaloric cooling process with a constant strain of 600% at room temperature.

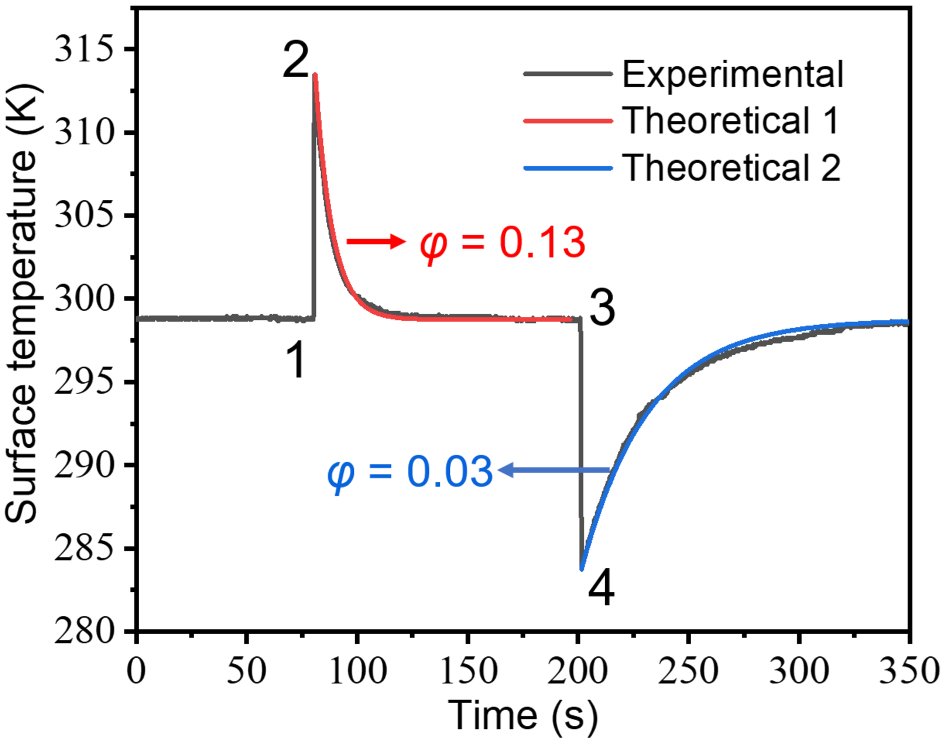

Supplementary Figure 5. Typical surface temperature variation of TPEs-1 as a function of elapsed time during single E-CE cycle. The black curve represents experimental values recorded using an on-line infrared thermometer. The red and blue curves represent theoretical values of isothermal process, which calculated by Newton's law of cooling.

Newton's law of cooling in Supplementary Fig. 5.

$$T = T_a + (T_0 - T_a)e^{-\varphi t} \tag{16}$$

Where T is the surface temperature (K) of specimen, T_a is the ambient temperature (K), T_0 is the initial surface temperature (K) of specimen, φ is a constant and t is the elapsed time (s). The difference of φ value between 2−3 and 4−1 process mainly comes from the change of surface area before and after deformation.

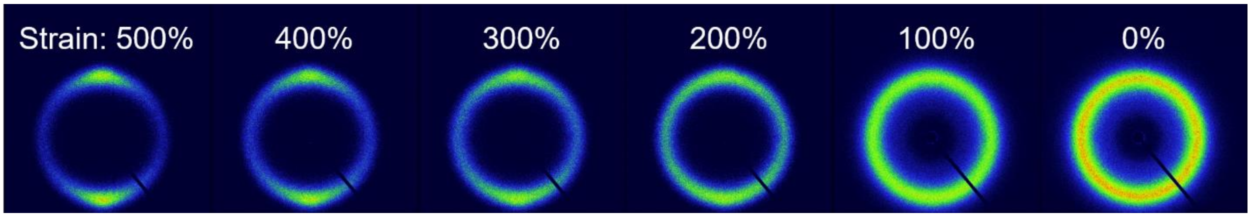

Supplementary Figure 6. 2D WAXD diffractograms of TPEs-1 at different strain levels during the recovery process.

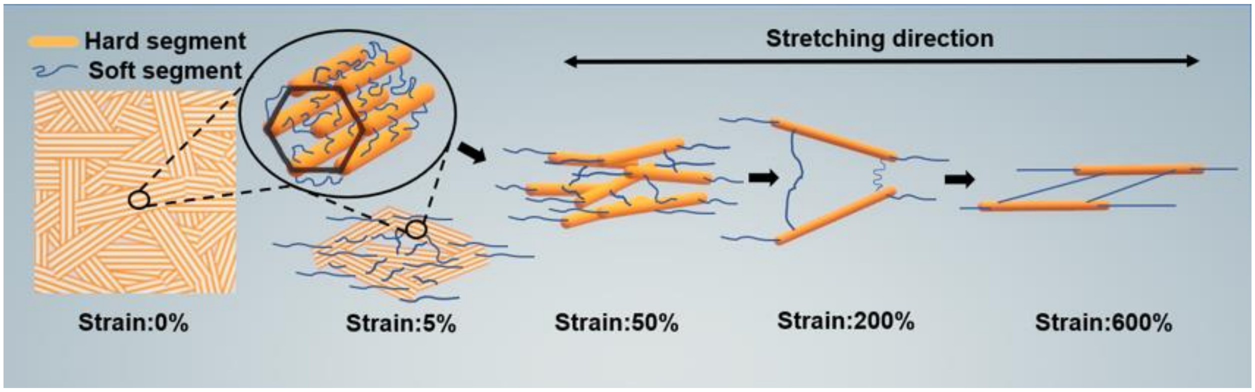

Supplementary Figure 7. Schematic diagram of aggregate structure evolution pathway in the stretching process of TPEs-1. The orange cylinders and blue fibers represent hard PS segments and PE/PB soft segments, respectively.

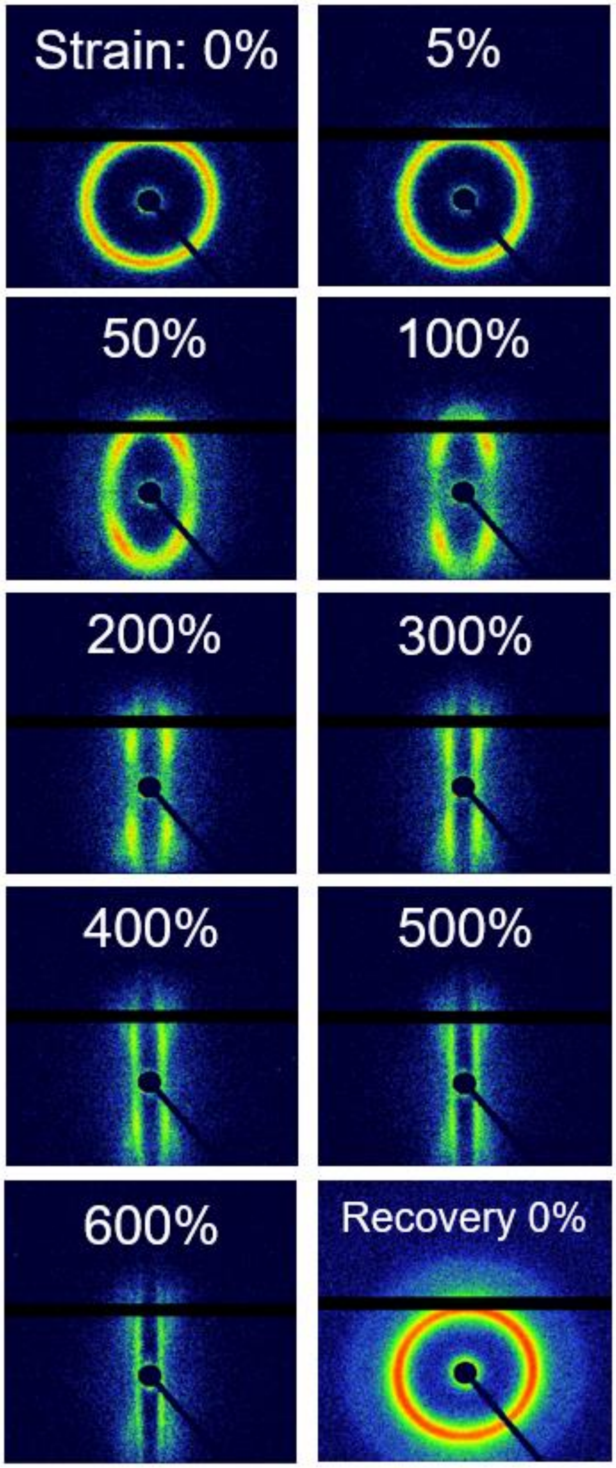

Supplementary Figure 8. 2D SAXS diffractograms of TPEs-1 at different strain levels during tensile recovery cycle at room temperature.

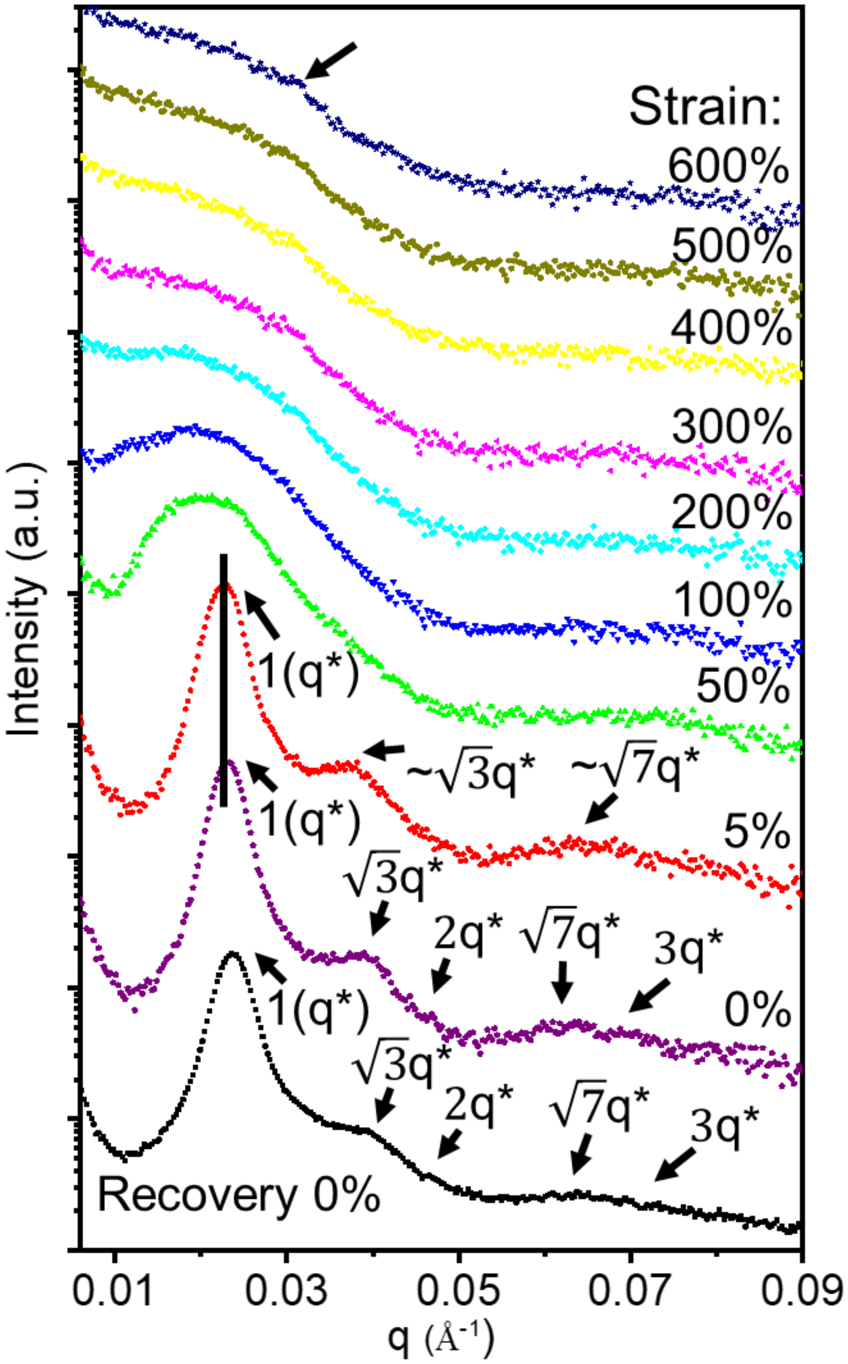

Supplementary Figure 9. 1D SAXS profiles at different strain levels obtained by full tilted circular integration of corresponding 2D SAXS patterns of TPEs-1.

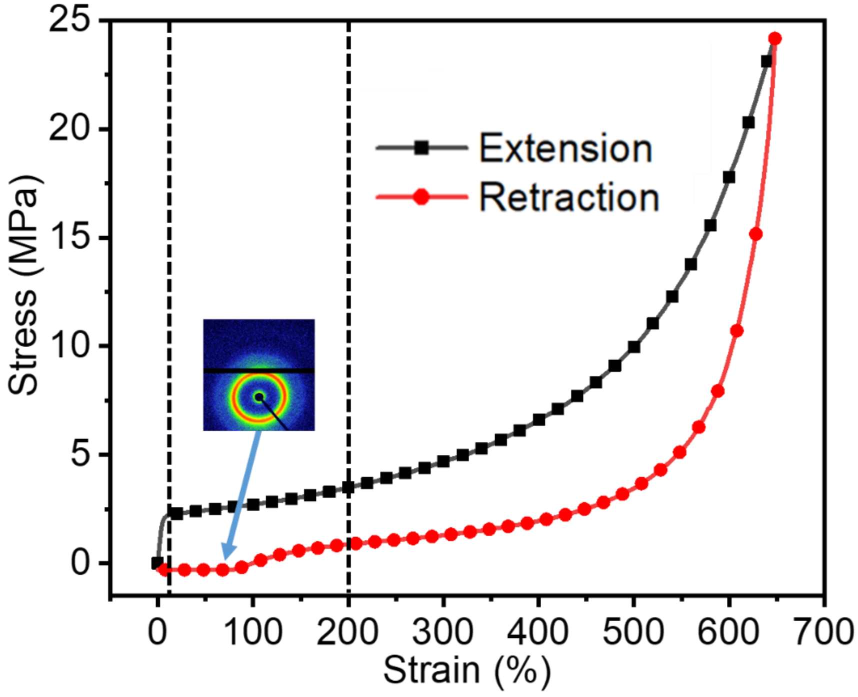

Supplementary Figure 10. The stress-strain curve during the first stretching and retraction cycle of TPEs-1 at room temperature.

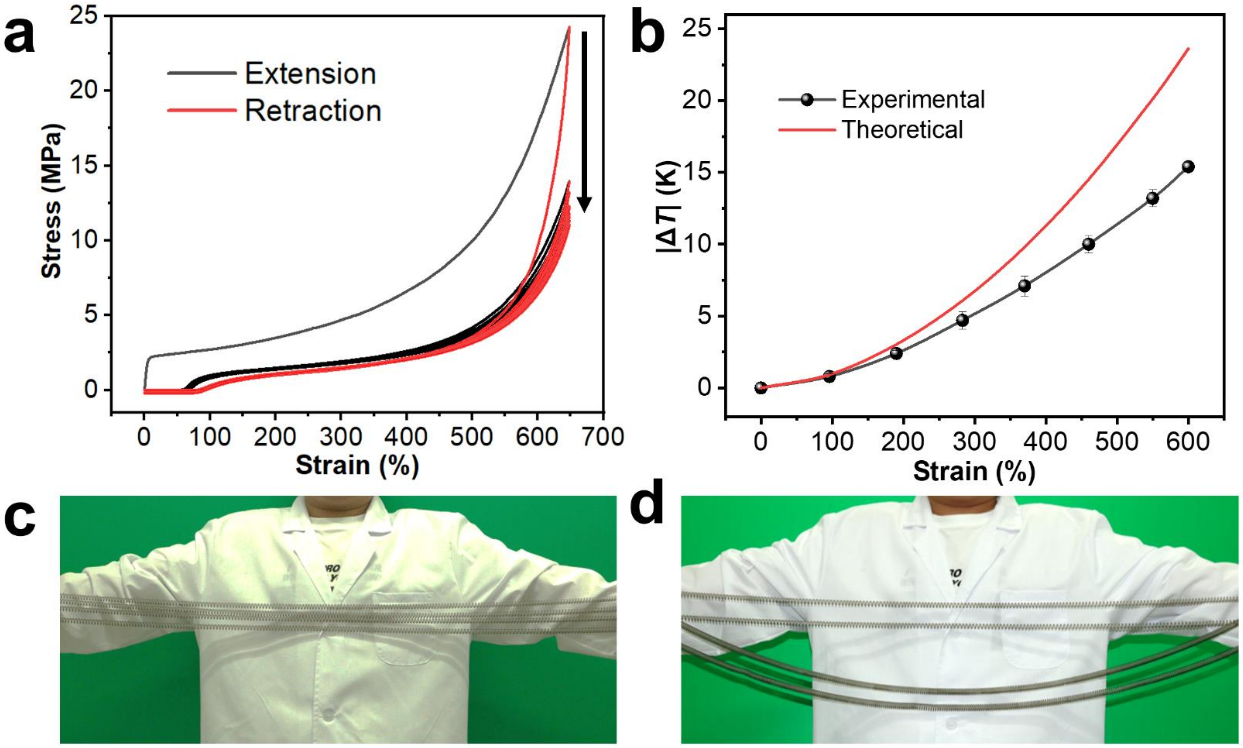

Supplementary Figure 11. Strain dependence of E-CE for TPEs-1. a The stress-strain curve of the sample TPEs-1 after ten stretching and recovery cycles. The black arrow points to the increasing number of cycles. **b** Experimental and theoretical temperature variation as a function of strain on cooling process. All tests were performed at room temperature. **c** Ideal stretching model of molecular chains with uniform chain-lengths. **d** Ideal stretching model of molecular chains with short chains and long chains.

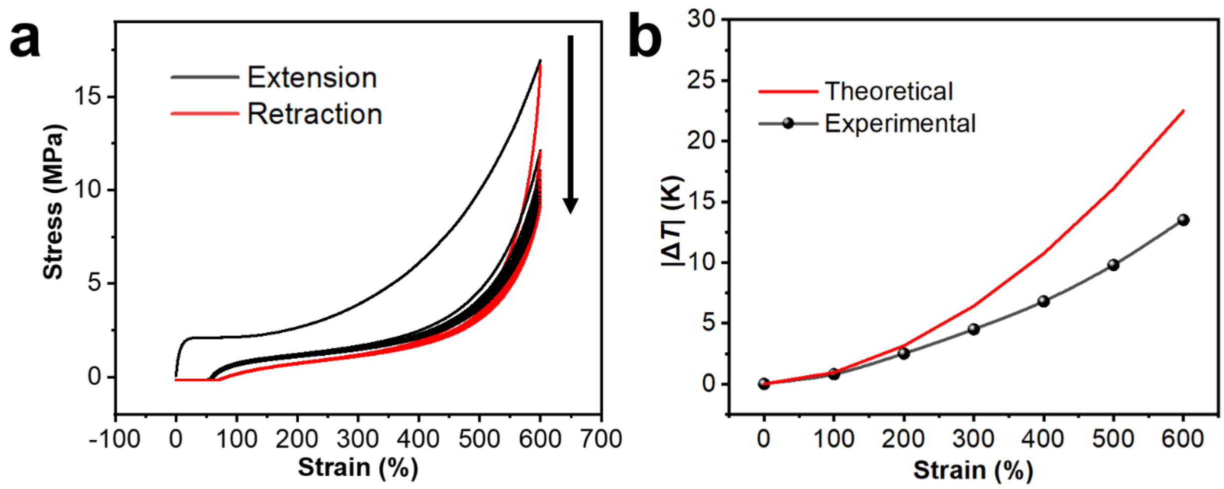

Supplementary Figure 12. Strain dependence of E-CE for TPEs-2. a The stress-strain curve of the TPEs-2 sample after ten stretching and recovery cycles. The black arrow points to the increasing number of cycles. **b** Experimental and theoretical temperature variation as a function of strain on cooling process. All tests were performed at room temperature.

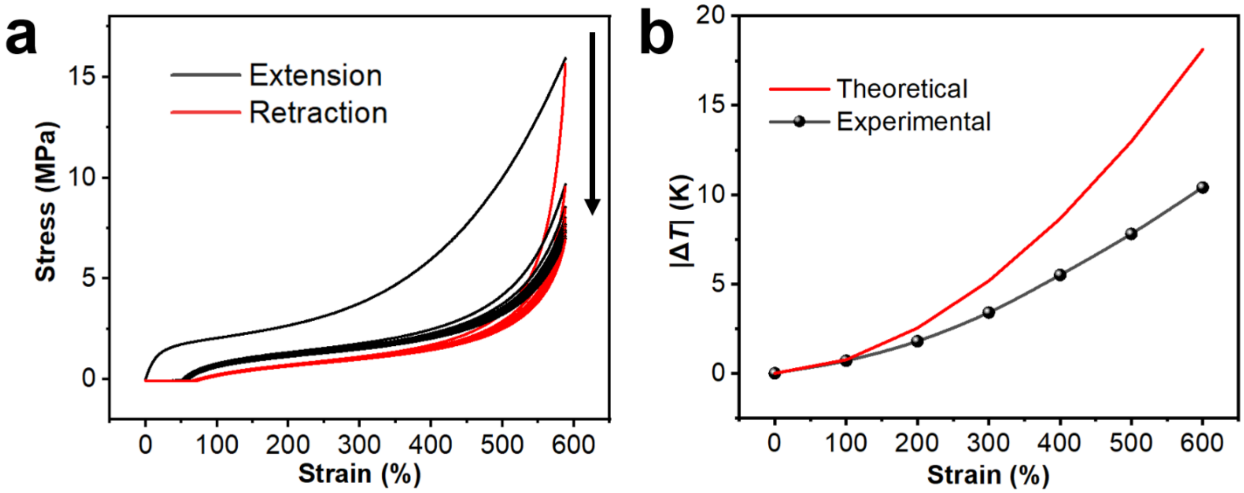

Supplementary Figure 13. Strain dependence of E-CE for TPEs-3. a The stress-strain curve of the TPEs-3 sample after ten stretching and recovery cycles. The black arrow points to the increasing number of cycles. **b** Experimental and theoretical temperature variation as a function of strain on cooling process. All tests were performed at room temperature.

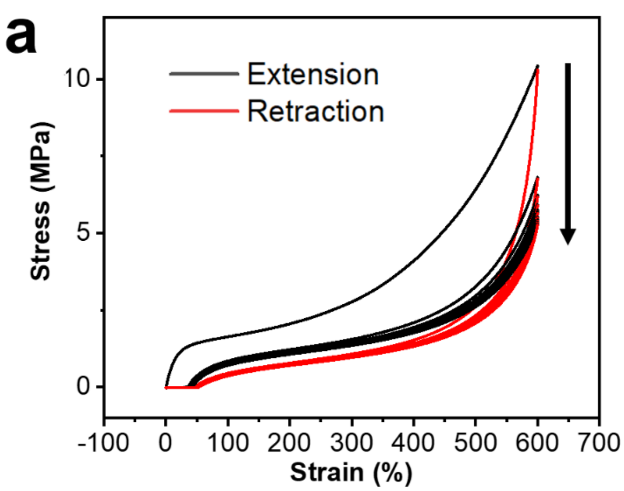

Supplementary Figure 14. Strain dependence of E-CE for TPEs-4. a The stress-strain curve of the TPEs-4 sample after ten stretching and recovery cycles. The black arrow points to the increasing number of cycles. **b** Experimental and theoretical temperature variation as a function of strain on cooling process. All tests were performed at room temperature.

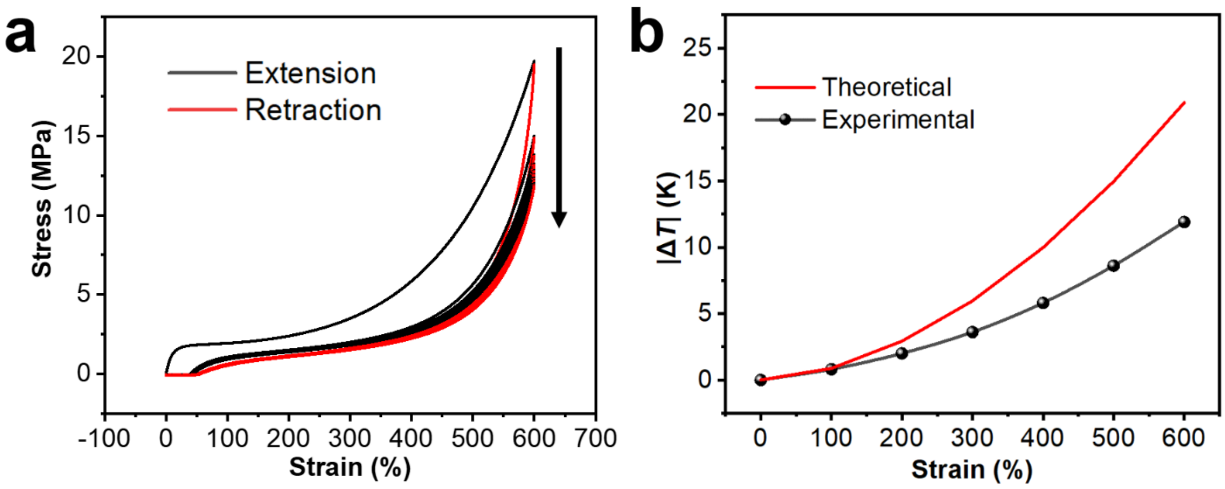

Supplementary Figure 15. Strain dependence of E-CE for TPEs-5. a The stress-strain curve of the TPEs-5 sample after ten stretching and recovery cycles. The black arrow points to the increasing number of cycles. **b** Experimental and theoretical temperature variation as a function of strain on cooling process. All tests were performed at room temperature.

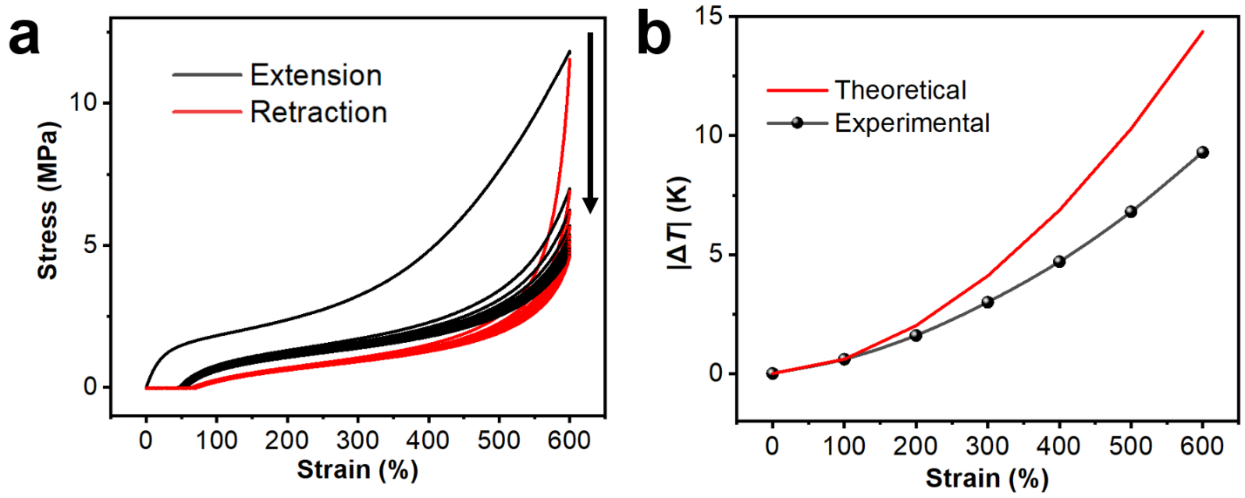

Supplementary Figure 16. Strain dependence of E-CE for TPEs-6. a The stress-strain curve of the TPEs-6 sample after ten stretching and recovery cycles. The black arrow points to the increasing number of cycles. **b** Experimental and theoretical temperature variation as a function of strain on cooling process. All tests were performed at room temperature.

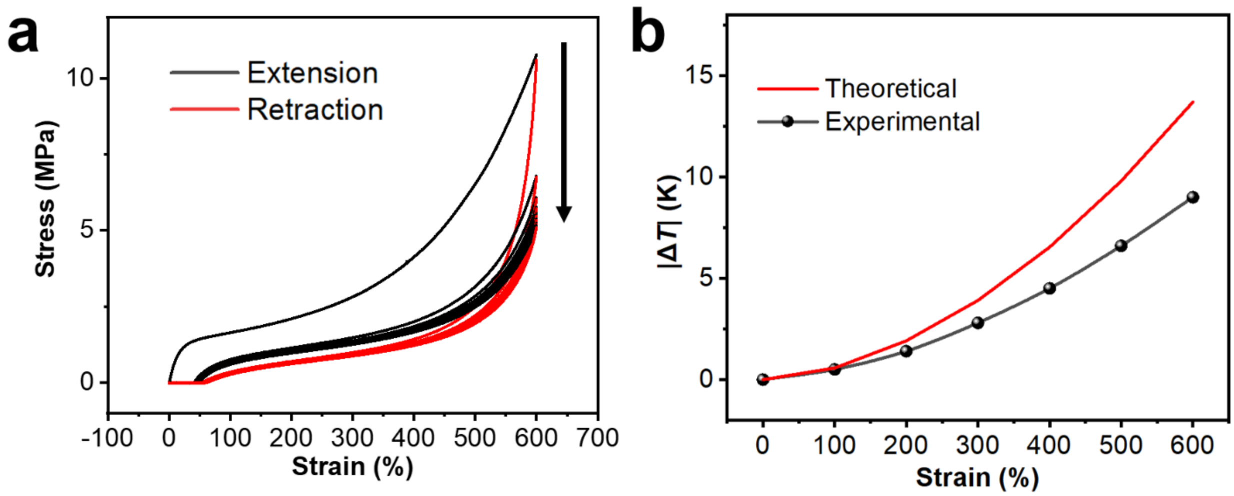

Supplementary Figure 17. Strain dependence of E-CE for TPEs-7. a The stress-strain curve of the TPEs-7 sample after ten stretching and recovery cycles. The black arrow points to the increasing number of cycles. **b** Experimental and theoretical temperature variation as a function of strain on cooling process. All tests were performed at room temperature.

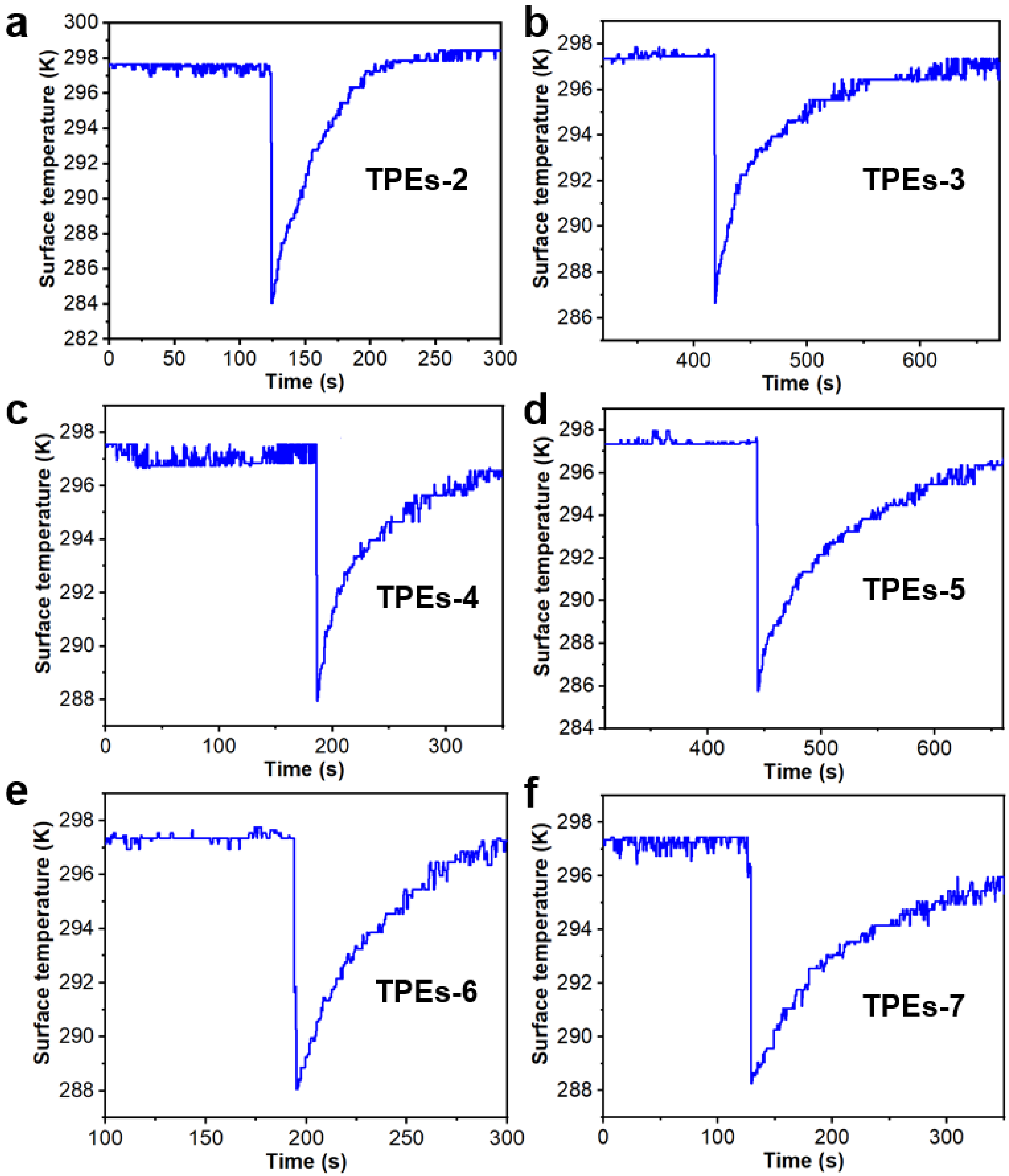

Supplementary Figure 18. Typical surface temperature variation of TPEs as a function of elapsed time during single E-CE cycle on cooling process.

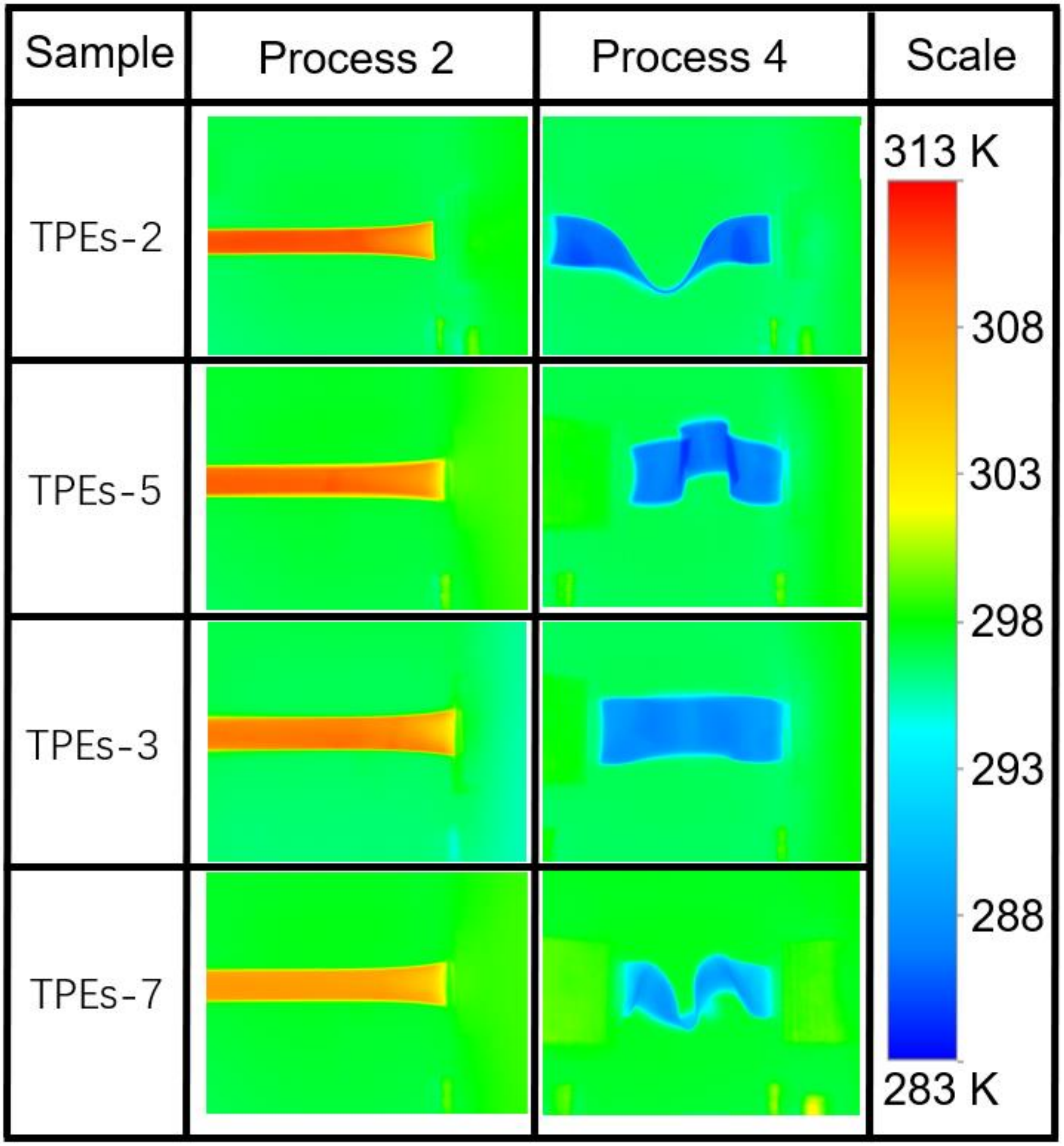

Supplementary Figure 19. Infrared thermal images of TPEs-2, TPEs-3, TPEs-5 and TPEs-7 at process nodes 2 and process nodes 4, recorded using an infrared thermal imager.

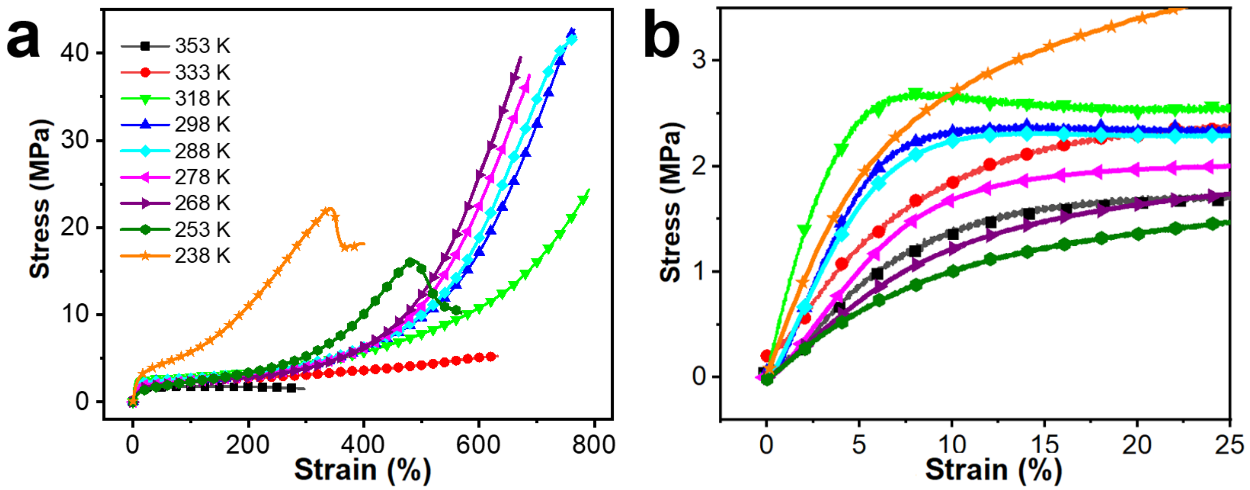

Supplementary Figure 20. a Stress-strain curves of TPEs-1 at different ambient temperature. **b** The magnification of the stress-strain curves in Supplementary Fig. 20a before 25% strain.

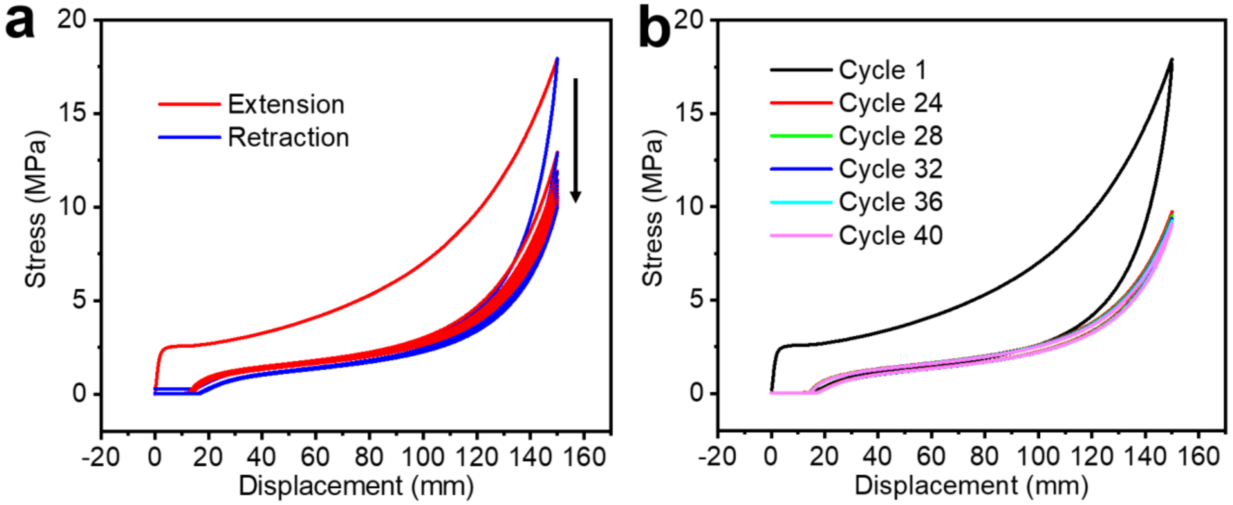

Supplementary Figure 21. a Stress-displacement characteristics of the TPEs-1 sample during the first 20 times of stretching and recovery cycles. The black arrow points to the increasing number of cycles. **b** Stress-displacement characteristics of the TPEs-1 sample after 40 times of stretching recovery cycles.

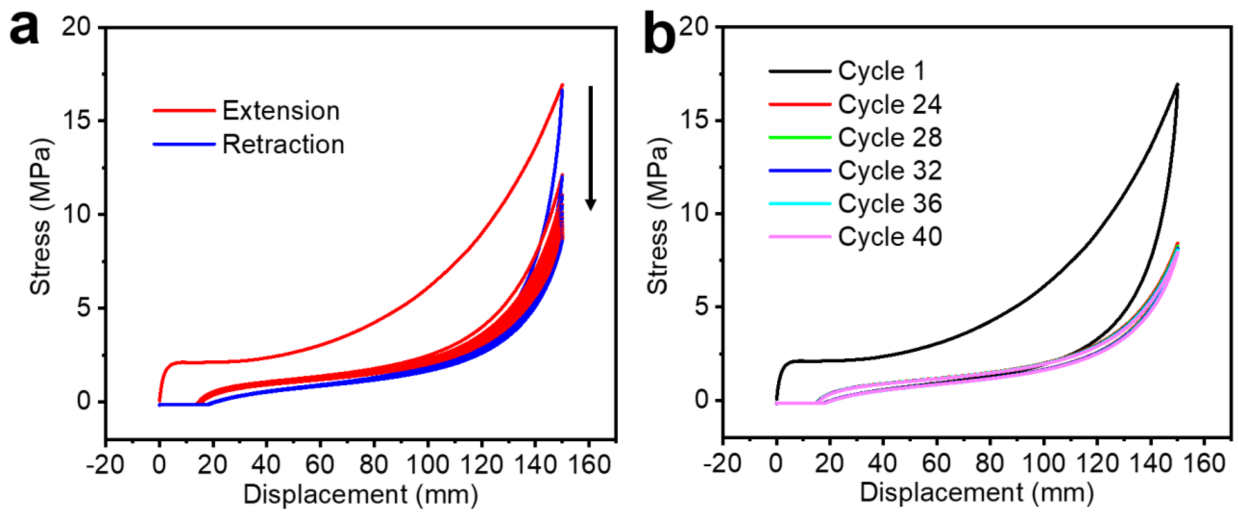

Supplementary Figure 22. a Stress-displacement characteristics of the TPEs-2 sample during the first 20 times of stretching and recovery cycles. The black arrow points to the increasing number of cycles. **b** Stress-displacement characteristics of the TPEs-2 sample after 40 times of stretching recovery cycles.

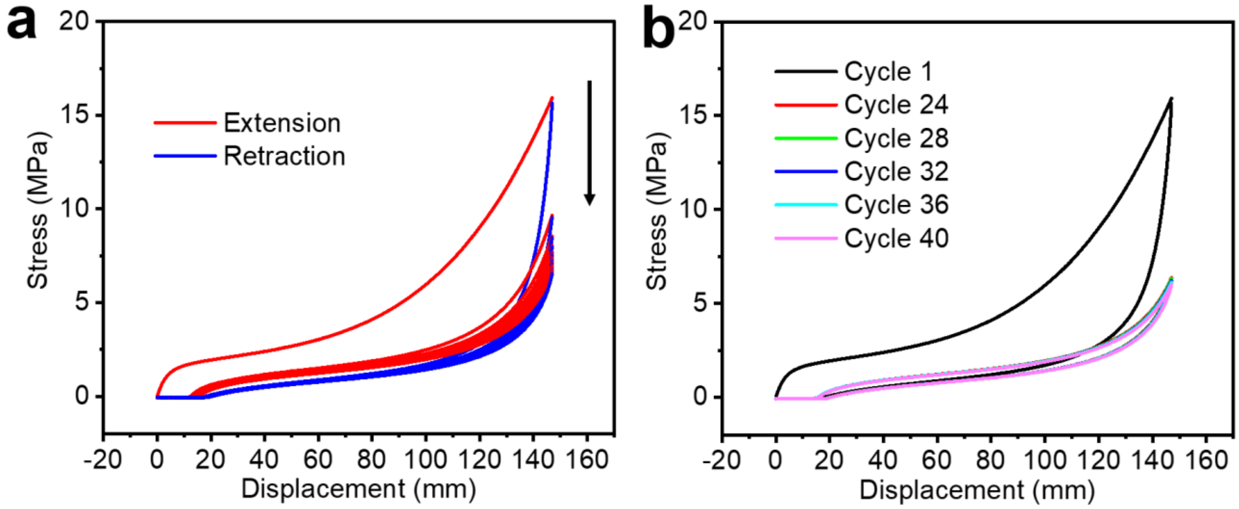

Supplementary Figure 23. a Stress-displacement characteristics of the TPEs-3 sample during the first 20 times of stretching and recovery cycles. The black arrow points to the increasing number of cycles. **b** Stress-displacement characteristics of the TPEs-3 sample after 40 times of stretching recovery cycles.

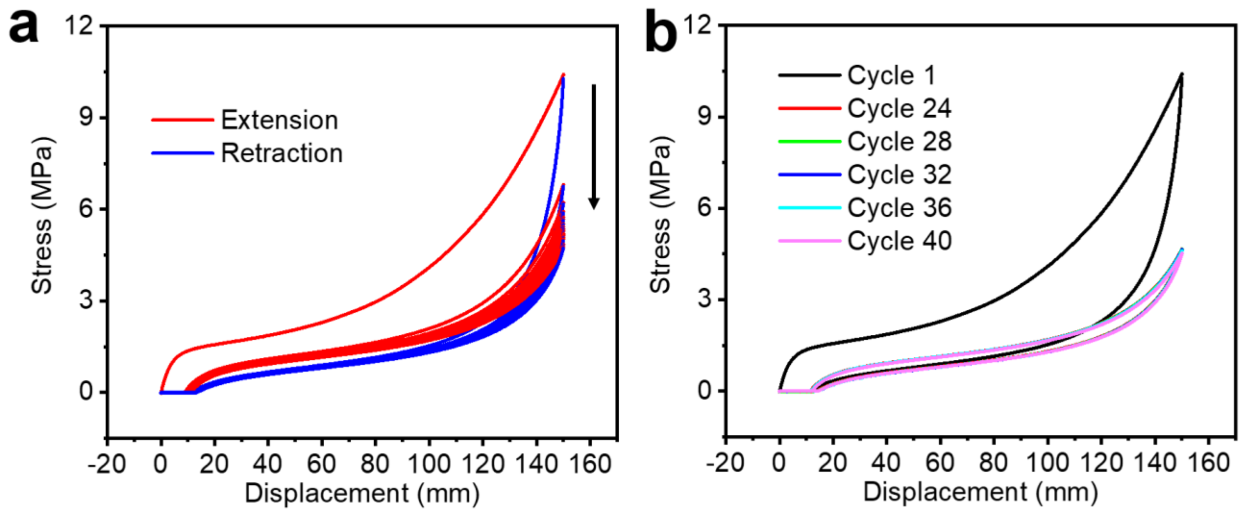

Supplementary Figure 24. a Stress-displacement characteristics of the TPEs-4 sample during the first 20 times of stretching and recovery cycles. The black arrow points to the increasing number of cycles. **b** Stress-displacement characteristics of the TPEs-4 sample after 40 times of stretching recovery cycles.

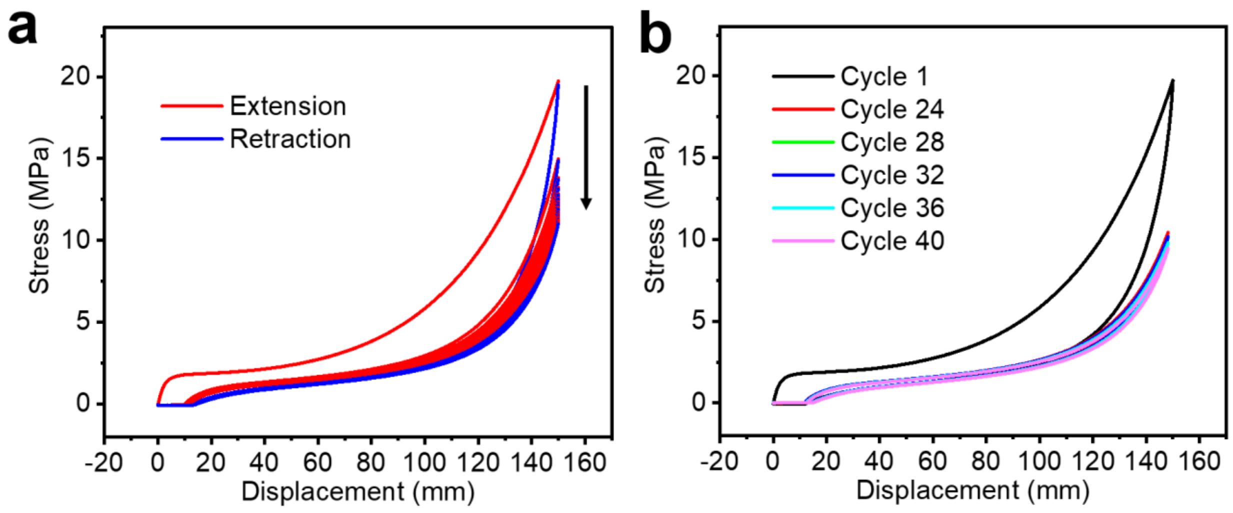

Supplementary Figure 25. a Stress-displacement characteristics of the TPEs-5 sample during the first 20 times of stretching and recovery cycles. The black arrow points to the increasing number of cycles. **b** Stress-displacement characteristics of the TPEs-5 sample after 40 times of stretching recovery cycles.

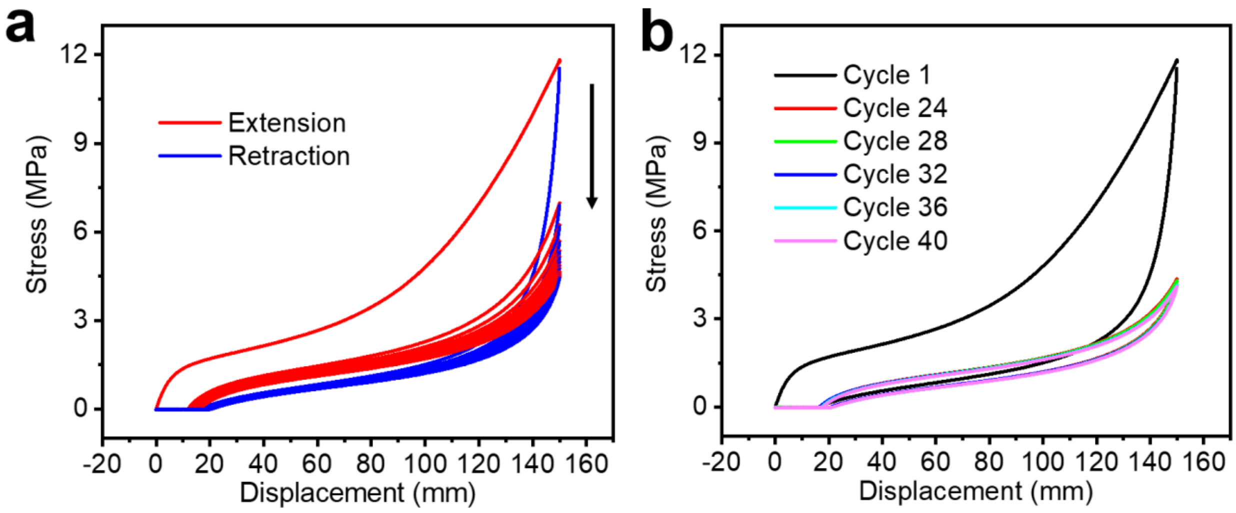

Supplementary Figure 26. a Stress-displacement characteristics of the TPEs-6 sample during the first 20 times of stretching and recovery cycles. The black arrow points to the increasing number of cycles. **b** Stress-displacement characteristics of the TPEs-6 sample after 40 times of stretching recovery cycles.

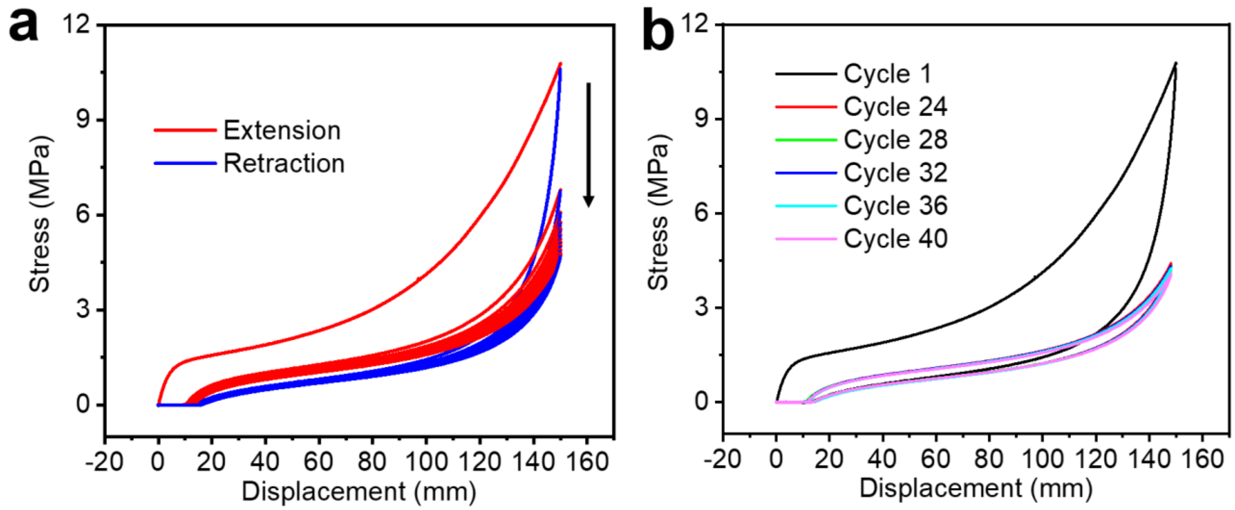

Supplementary Figure 27. a Stress-displacement characteristics of the TPEs-7 sample during the first 20 times of stretching and recovery cycles. The black arrow points to the increasing number of cycles. **b** Stress-displacement characteristics of the TPEs-7 sample after 40 times of stretching recovery cycles.

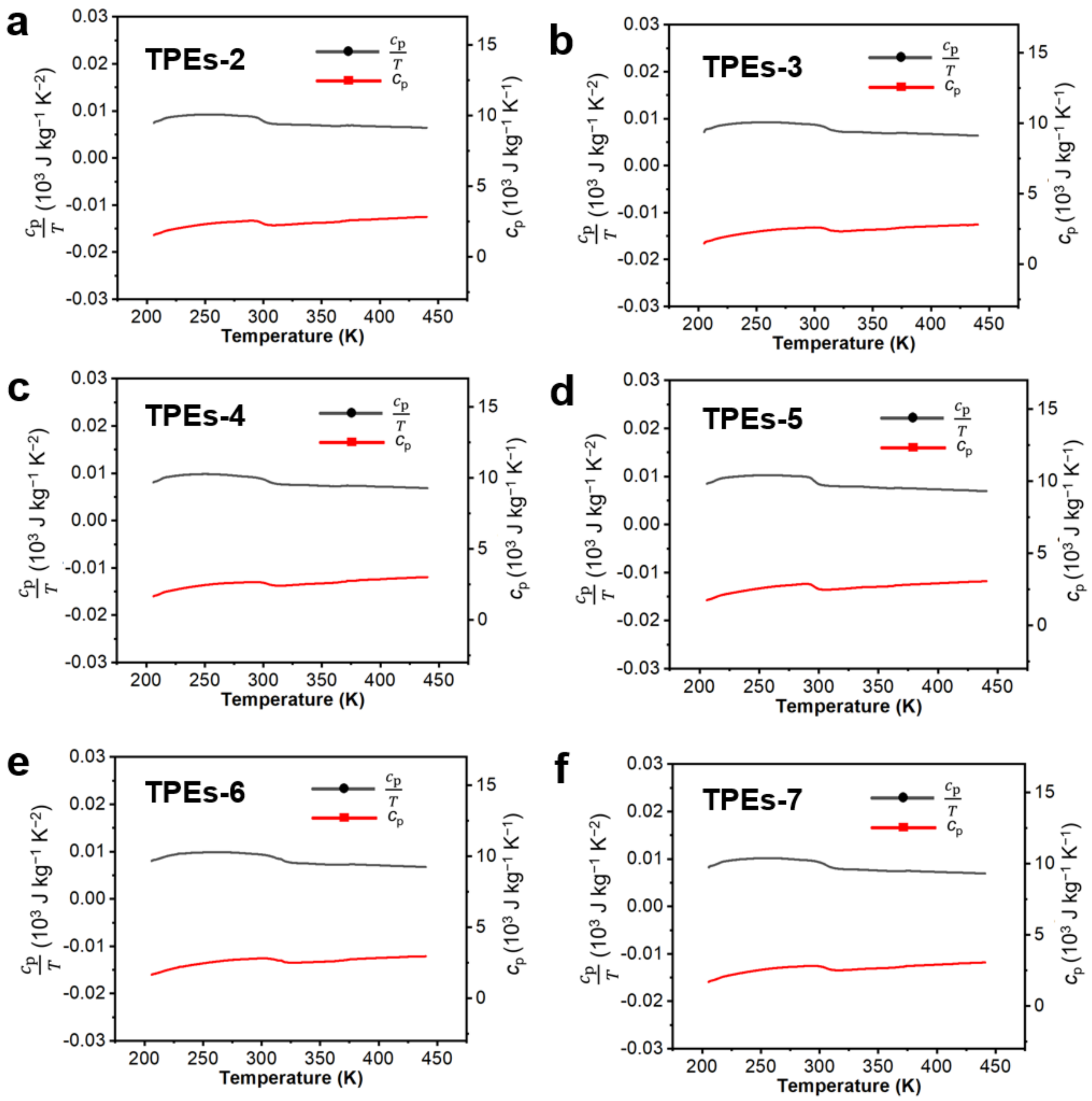

Supplementary Figure 28. Heat capacity c_p and $\frac{c_p}{T}$ versus temperature of TPEs.

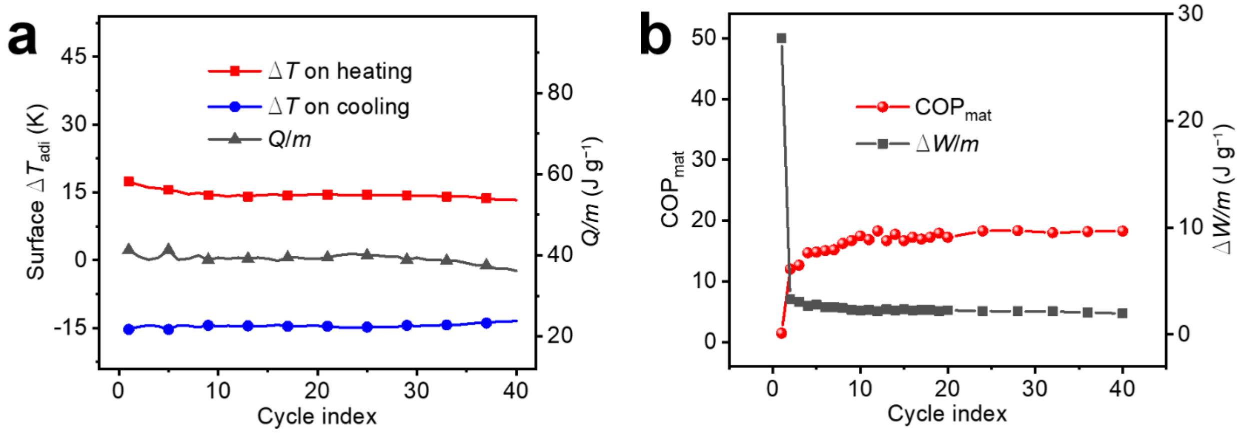

Supplementary Figure 29. Cyclic behaviors of E-CE for TPEs-1. a Surface ΔT_{adi} evolution of TPEs-1 during 40 times of E-CE cycles. **b** COP_{mat} and input work of unit mass in TPEs-1 during E-CE cycles.

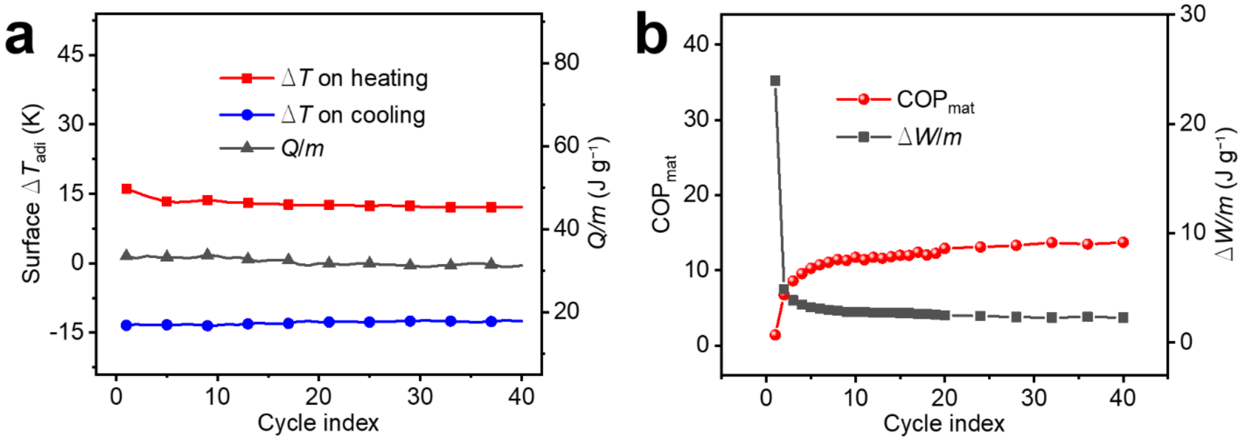

Supplementary Figure 30. Cyclic behaviors of E-CE for TPEs-2. a Surface ΔT_{adi} evolution of TPEs-2 during 40 times of E-CE cycles. **b** COP_{mat} and input work of unit mass in TPEs-2 during E-CE cycles.

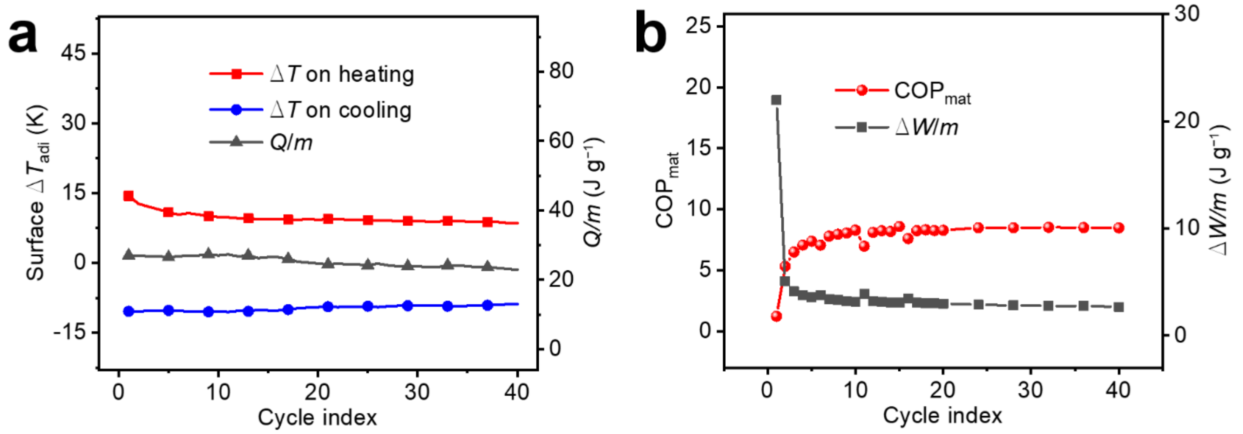

Supplementary Figure 31. Cyclic behaviors of E-CE for TPEs-3. a Surface ΔT_{adi} evolution of TPEs-3 during 40 times of E-CE cycles. **b** COP_{mat} and input work of unit mass in TPEs-3 during E-CE cycles.

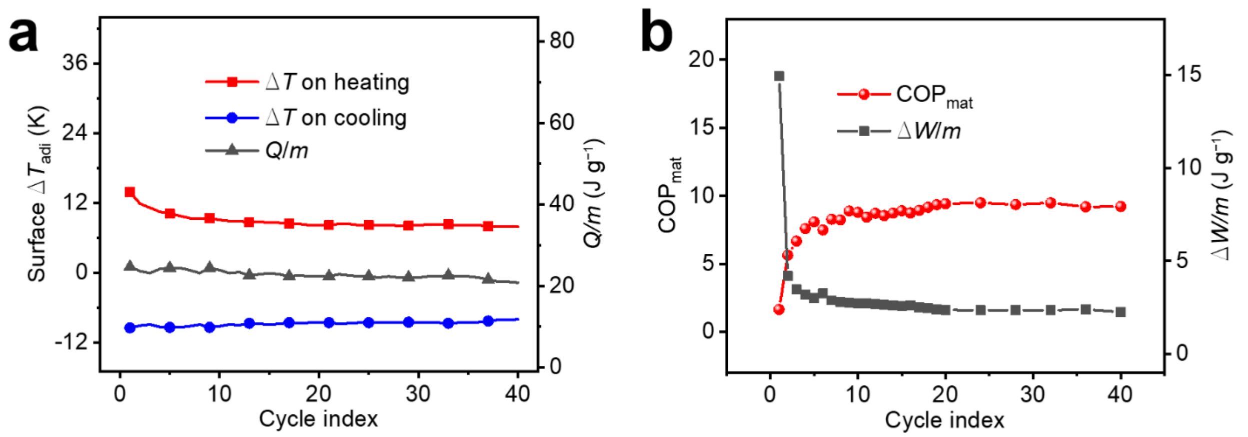

Supplementary Figure 32. Cyclic behaviors of E-CE for TPEs-4. a Surface ΔT_{adi} evolution of TPEs-4 during 40 times of E-CE cycles. **b** COP_{mat} and input work of unit mass in TPEs-4 during E-CE cycles.

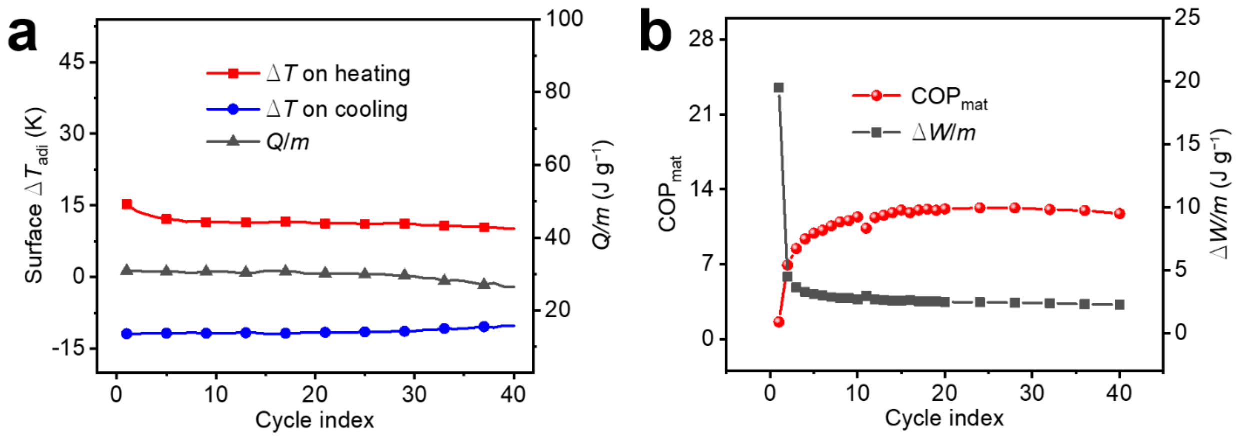

Supplementary Figure 33. Cyclic behaviors of E-CE for TPEs-5. a Surface ΔT_{adi} evolution of TPEs-5 during 40 times of E-CE cycles. **b** COP_{mat} and input work of unit mass in TPEs-5 during E-CE cycles.

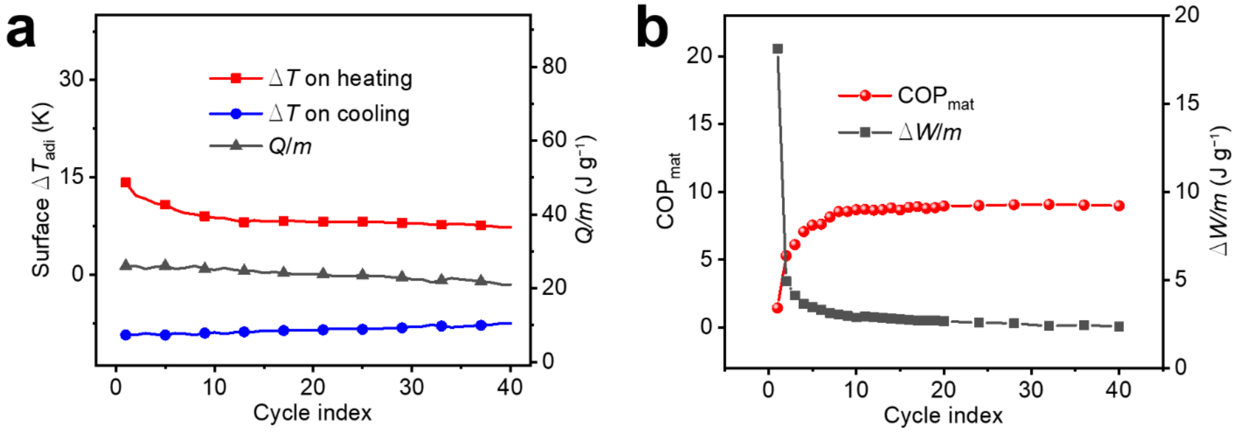

Supplementary Figure 34. Cyclic behaviors of E-CE for TPEs-6. a Surface ΔT_{adi} evolution of TPEs-6 during 40 times of E-CE cycles. **b** COP_{mat} and input work of unit mass in TPEs-6 during E-CE cycles.

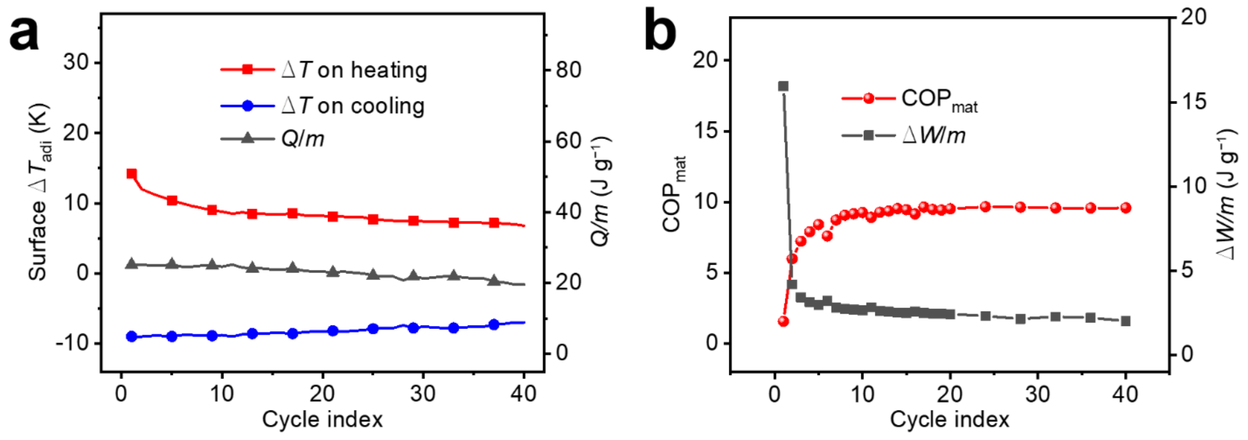

Supplementary Figure 35. Cyclic behaviors of E-CE for TPEs-7. a Surface ΔT_{adi} evolution of TPEs-7 during 40 times of E-CE cycles. **b** COP_{mat} and input work of unit mass in TPEs-7 during E-CE cycles.

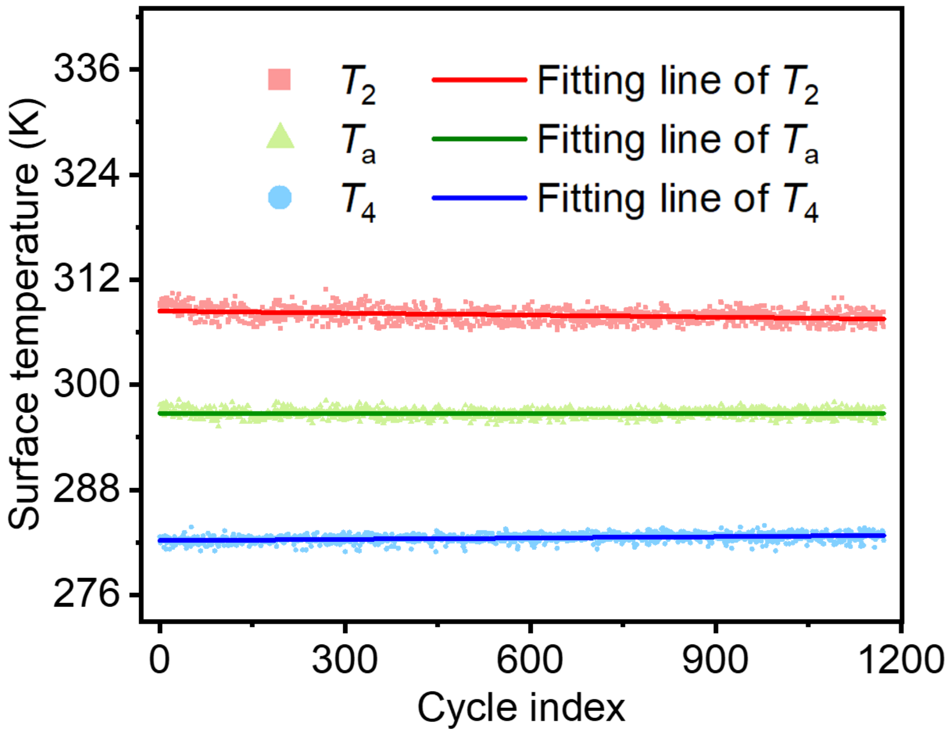

Supplementary Figure 36. Surface temperature evolution of TPEs-1 during 1000 times of E-CE cycles. The strain rate of the extension process was set as 1 s^{-1}. The strain rate of the retraction process was set as 15 s^{-1}. The T_a temperature is the average value of the ambient temperature corresponding to each cycle. The fitting lines were obtained by linear fitting of the T_2, T_a and T_4 data points.

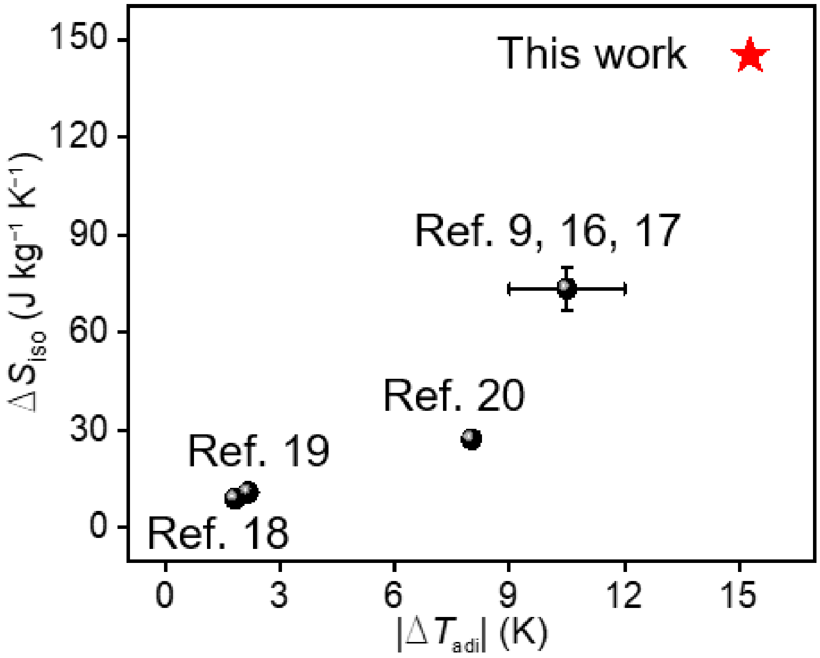

Supplementary Figure 37. $|\Delta T_{adi}|$ and ΔS_{iso} comparison of TPEs-1 with reported elastocaloric polymers.

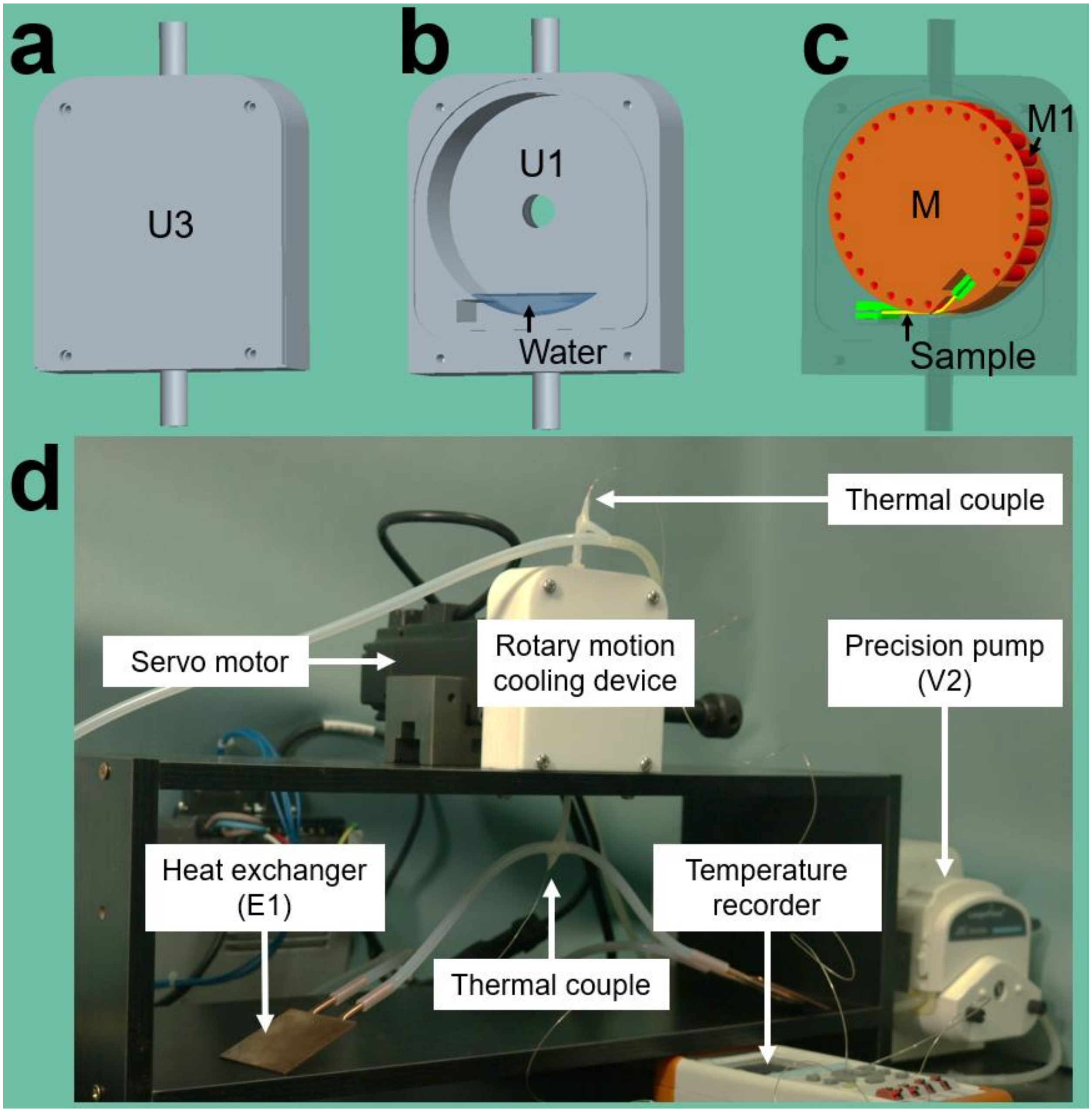

Supplementary Figure 38. Schematic and physical illustration of the rotary motion elasto-based cooling device. a Rotary motion elasto-based cooling device after the installation of cover plate. **b** Internal contour of body case. **c** The structure of revolving disc and clips. **d** The physical picture of the cooling device and circulation system.

Supplementary Table 1. Comparison of various elastocaloric materials on the $|\Delta S_{iso}|$, $|\Delta T_{adi}|$, $|\sigma|$, strain ($|\varepsilon|$) and COP_{mat} near room temperature.

Compound	$|\Delta S_{iso}|$ (J kg^{-1} K^{-1})	$|\Delta T_{adi}|$ (K)	$|\sigma|$ (MPa)	$|\varepsilon|$ (%)	COP_{mat}	Ref.
$Ni_{55.92}Ti_{44.08}$	-	20	1150	3.7	5	21
$Ti_{50.5}Ni_{49.1}Fe_{0.4}$	-	18.1	610	6.3	2.5	22
$(Ni_{50}Mn_{31.5}Ti1_{8.5})_{99.8}B_{0.2}$	45	31.5	700	7	-	23
$Ni_{54}Fe_{19}Ga_{27}Co_{4}$	11	10	300	5	14	24
$Co_{40}Ni_{33}Al_{27}$	44	3	50	10	-	25
SEBS	145	15.3	12	600	16.2	This work
NR	77	9-12	1-2	600	-	9,16,17
PVDF-TrFE-CTFE	9	1.8	15	2.5	-	18
PVDF-TrFE-CTFE	11	2.15	-	12	-	19
Polyurethane	27.3	8	6.5	400	8	20
Li_3N	20	2	5800	2	-	26

Supplementary Table 2. Samples labeling and molecular properties of SEBS

Sample	$\overline{M}$n (10^4 g mol^{-1})	$\overline{M}$w (10^4 g mol^{-1})	$M_{w\text{-}max}$ (10^4 g mol^{-1})	$M_{w\text{-}min}$ (10^4 g mol^{-1})	MR [a] (10^4 g mol^{-1})
TPEs-1	5.9	7.6	13.3	2.9	10.4
TPEs-2	14.2	15.6	22.8	8.3	14.5
TPEs-3	16.7	20.8	41.3	8.8	32.5
TPEs-4	25.4	30.8	13.5	56.7	43.2
TPEs-5	29.0	31.8	18.2	47.1	28.9
TPEs-6	29.8	35.0	16.3	62.3	44.9
TPEs-7	36.4	41.4	25.4	77.9	52.5

[a]. The molecular weight distribution range (MR) is defined as the difference value of the maximum molecular weight ($M_{w\text{-}max}$) and the minimum molecular weight ($M_{w\text{-}min}$) of a sample. For the convenience of quantification, the molecular weights with the integral distribution (Ht) of 0.05 and 0.95 on the integral distribution curve was regarded as $M_{w\text{-}min}$, and $M_{w\text{-}max}$ (Fig. 2E and Supplementary Fig. 1) respectively. The molecular weight presented in this paper is from the whole macromolecular chains, including PS and PE/PB segments in a single macromolecular chain. In the process of anionic polymerization, the molecular weight distribution of PS is relatively easy to control. Therefore, it can be considered that the heterogeneity of molecular weight of the integrated macromolecular chains mainly comes from the PE/PB matrix blocked in the molecular chains.

The product names of TPEs-1 and TPEs-5 were G1650MV and G1651EU, respectively. These samples were provided by KRATON Polymers Co., Ltd. (http://www.kraton-polymers.cn/). The product names of TPEs-2, TPEs-3, TPEs-4 and TPEs-7 were 6154, 6153, 6151 and 6159, respectively. These samples were provided by TSRC Industries Co., Ltd. (https://www.tsrc.com.tw/). The product name of TPEs-6 was 503. This sample was provided by Sinopec Group Co., Ltd. (http://www.sinopec.com/).

Supplementary Table 3. The modulus of TPEs obtained from Supplementary Fig. 11 to Supplementary Fig. 17

Sample	E (MPa)	MR (10^4 g mol^{-1})	$\lvert\Delta T_{adi}\rvert$ (K)
TPEs-1	4.22	10.4	15.3
TPEs-2	3.64	14.5	13.5
TPEs-3	3.06	32.5	10.6
TPEs-4	2.75	43.2	9.5
TPEs-5	3.52	28.9	11.7
TPEs-6	2.60	44.9	9.3
TPEs-7	2.49	52.5	9

Supplementary Table 4. SCP_{sys} comparison of cooling systems based on different caloric effects (CEs).

CEs	Material	Sample shape	SCP_{sys} ($W\ g^{-1}$)	Ref.
Magnetocaloric	NdFeB	Metal film	2	27
	Gadolinium	Metal ball	0.17	28
Electrocaloric	P(VDF-TrFE-CFE)	Polymer multilayer	2.8	29
	P(VDF-TrFE-CFE)	Polymer multilayer	3.6	30
	$PbSc_{0.5}Ta_{0.5}O_3$	Ceramic multilayer	0.007	31
	$Pb(Sc,Ta)O_3$	Ceramic multilayer	0.85	32
Elastocaloric	NR	Polymer balloon	20.9	33
	SEBS	Polymer film	1.9	This work
	$Ni_{0.558}Ti_{0.442}$	Metal sheet	0.8	34
	Ni-Ti	Metal wire	0.25	35

Supplementary References

1. Wijayasekara, D. B., Huang, T. Z., Richardson, J. M., Knauss, D. M. & Bailey, T. S. The role of architecture in the melt-state self-assembly of (polystyrene)star-b-(polyisoprene)linear-b-(polystyrene)star pom-pom triblock copolymers. *Macromolecules* **49**, 595-608 (2016).
2. Li, T., Senesi, A. J. & Lee, B. Small Angle X-ray Scattering for Nanoparticle Research. *Chem. Rev.* **116**, 11128–11180 (2016).
3. Svergun, D. I. & Koch, M. H. J. Small-angle scattering studies of biological macromolecules in solution. *Rep. Prog. Phys.* **66**, 1735–1782 (2003).
4. Stern-Taulats, E. et al. Magnetocaloric effect in the low hysteresis Ni-Mn-In metamagnetic shape-memory Heusler alloy. *J. Appl. Phys.* **115**, 173907 (2014).
5. Casanova, F. et al. Direct observation of the magnetic-field-induced entropy change in Gd5(SixGe1−x)4 giant magnetocaloric alloys. *Appl. Phys. Lett.* **86**, 262504 (2005).
6. Moya, X. et al. Giant electrocaloric strength in single-crystal BaTiO3. *Adv. Mater.* **25**, 1360–1365 (2013).
7. Valant, M. Electrocaloric materials for future solid-state refrigeration technologies. *Prog. Mater. Sci.* **57**, 980-1009 (2012).
8. Lloveras, P. et al. Colossal barocaloric effects near room temperature in plastic crystals of neopentylglycol. *Nat. Commun.* **10**, 1803 (2019).
9. Xie, Z. J., Sebald, G. & Guyomar, D. Comparison of direct and indirect measurement of the elastocaloric effect in natural rubber. *Appl. Phys. Lett.* **108**, 041901 (2016).
10. Wall, F. T. Statistical Thermodynamics of Rubber. III. *J. Chem. Phys.* **11**, 527-530 (1943).
11. Hua, Y. *Polymer Physics* (Chemical Industry Press, Peking, 2019).
12. Flory, P. J. & Rehner, J. Statistical Mechanics of Cross-Linked Polymer Networks I. Rubberlike

Elasticity. *J. Chem. Phys.* **11**, 512-520 (1943).

13. Haward, R. N. Strain Hardening of Thermoplastics. *Macromolecules* **26**, 5860-5869 (1993).
14. Liu, Y. et al. Effective of physical and chemical crosslinking structure on fatigue behavior of styrene butadiene elastomer. *J. Appl. Polym. Sci.* **131,** 40917 (2014).
15. Daniel R. B., Erika, G. H. & Ignacio, M. G. Impressive fatigue life and fracture toughness improvements in graphene oxide/epoxy composites. *Macromolecules* **45,** 238-245 (2012).
16. Xie, Z. J., Wei, C., Guyomar, D. & Sebald, G. Validity of Flory's model for describing equilibrium strain-induced crystallization (SIC) and thermal behavior in natural rubber. *Polymer* **103**, 41-45 (2016).
17. Guyomar, D. et al. Elastocaloric modeling of natural rubber. *Appl. Therm. Eng.* **57**, 33-38 (2013).
18. Patel, S., Chauhan, A., Vaish, R. & Thomas, P. Elastocaloric and barocaloric effects in polyvinylidene di-fluoride-based polymers. *Appl. Phys. Lett.* **108**, 072903 (2016).
19. Yoshida, Y., Yuse, K., Guyomar, D., Capsal, J. F. & Sebald, G. Elastocaloric effect in poly(vinylidene fluoride-trifluoroethylene-chlorotrifluoroethylene) terpolymer. *Appl. Phys. Lett.* **108**, 242904 (2016).
20. Coativy, G. et al. Elastocaloric properties of thermoplastic polyurethane. *Appl. Phys. Lett.* **117**, 193903 (2020).
21. Porenta, L. et al. Thin-walled Ni-Ti tubes under compression ideal candidates for efficient and fatigue-resistant elastocaloric cooling. *Appl. Mater. Today* **20**, 100712 (2020).
22. Ulpiani, G. et al. Upscaling of SMA film-based elastocaloric cooling. *Appl. Therm. Eng.* **180**, 115867 (2020).
23. Cong, D. Y. et al. Colossal Elastocaloric Effect in Ferroelastic Ni-Mn-Ti Alloys. *Phys. Rev.*

Lett. **122**, 255703 (2019).

24. Xiao, F., Jin, M., Liu, J. & Jin, X. Elastocaloric effect in $Ni_{50}Fe_{19}Ga_{27}Co_4$ single crystals. *Acta Mater.* **96,** 292-300 (2015).

25. Pataky, G. J., Ertekin, E. & Sehitoglu, H. Elastocaloric cooling potential of NiTi, Ni_2FeGa, and CoNiAl. *Acta Mater.* **96,** 420-427 (2015).

26. Sagotra, A. K., Chu, D. & Cazorla, C. Room-temperature mechanocaloric effects in lithium-based superionic materials. *Nat. Commun.* **9,** 3337 (2018).

27. Jacobs, S. et al. The performance of a large-scale rotary magnetic refrigerator. *Int. J. Refrig.* **37,** 84-91 (2014).

28. Aprea, C., Greco, A., Maiorino, A. & Masselli, C. The energy performance of a rotary permanet magnet magnetic refrigerator. *Int. J. Refrig.* **61,** 1-11 (2016).

29. Ma, R. J. et al. Highly efficient electrocaloric cooling with electrostatic actuation. *Science* **357**, 1130–1134 (2017).

30. Bo, Y. et al. Electrostatic actuating double-unit electrocaloric cooling device with high efficiency. *Adv. Energy Mater.* 2003771 (2021).

31. Wang, Y. et al. A high-performance solid-state electrocaloric cooling system. *Science* **370,** 129-133 (2020).

32. Torello, A. et al. Giant temperature span in electrocaloric regenerator. *Science* **370,** 125-129 (2020).

33. Greibich, F. et al. Elastocaloric heat pump with specific cooling power of 20.9 W g–1 exploiting snap-through instability and strain-induced crystallization. *Nat. Energy* **6**, 260-267 (2021).

34. Tusek, J. et al. A regenerative elastocaloric heat pump. *Nat. Energy* **1**, 16134 (2016).

35. Qian, S. X., Wang, Y., Yuan, L. F. & Yu, J. L. A heat driven elastocaloric cooling system. *Energy* **182**, 881-899 (2019).
